# Supplementary material for: Motion impact score for detecting spurious brain-behavior associations
Source: Nat Commun. 2025 Sep 29;16:8614. doi: 10.1038/s41467-025-63661-2 (PMC12479937; doi:10.1038/s41467-025-63661-2)
Supplement: Supplementary file 1 — Supplementary Information [file 41467_2025_63661_MOESM1_ESM.pdf]

Supplementary Figures

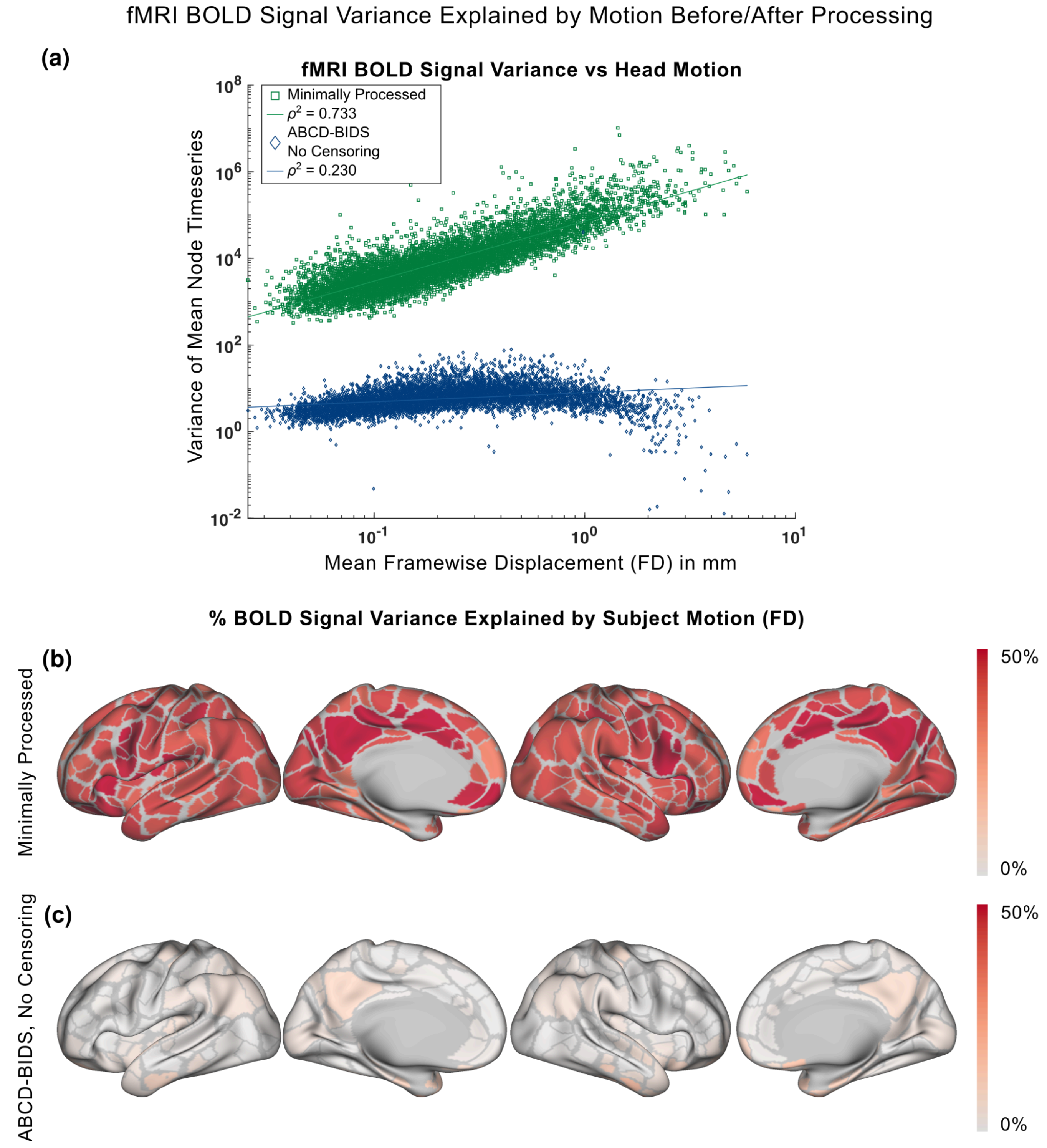

Supplementary Figure 1: Proportion of fMRI BOLD signal variance explained by head motion before and after processing with the ABCD-BIDS (DCAN Labs) motion reduction algorithm. The comparison is made prior to any framewise motion censoring. **(a)** Log-log plot of variance of the mean node timeseries *vs.* mean framewise displacement (FD, in mm) for each child (n = 9,652). The square of Spearman's rho, ρ^2 is provided for the log-log best fit. **(b)** Proportion of variance of mean node (parcel) timeseries explained by motion (Spearman ρ^2, log-log fit) visualized on the cortical surface before and **(c)** after ABCD-BIDS.

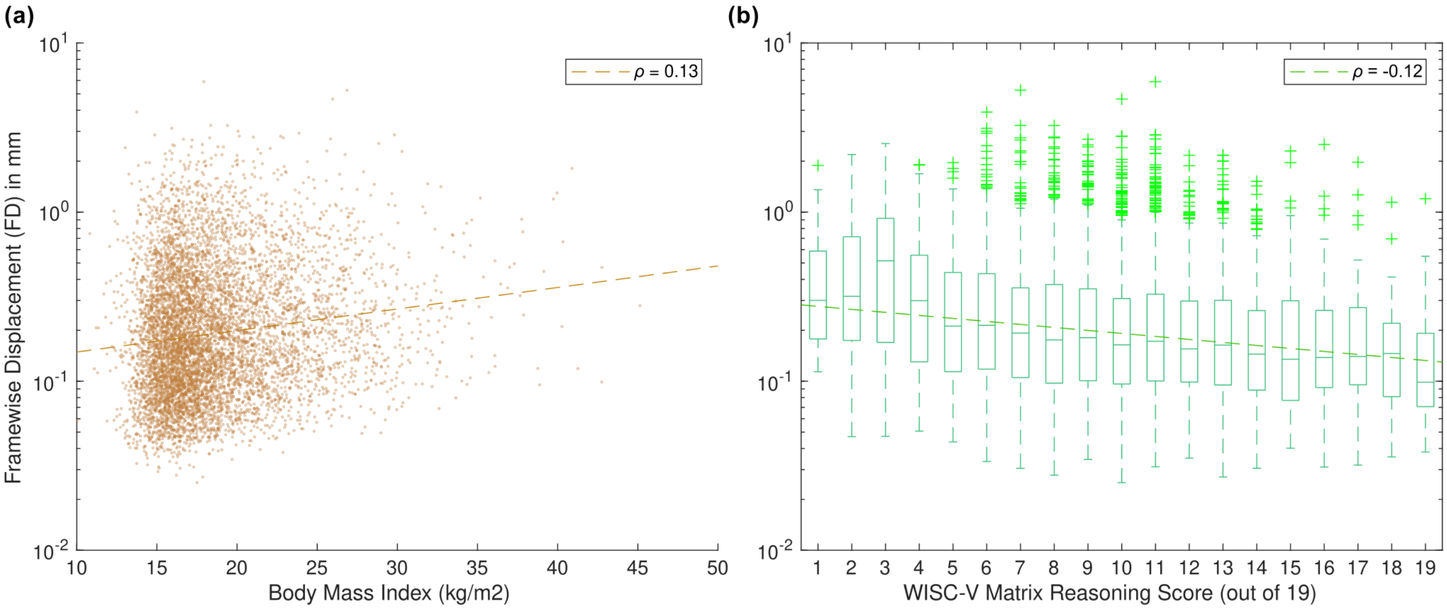

Supplementary Figure 2: Correlation between trait variables and in-scanner head motion. (a) Body mass index (BMI, kg/m^2, mean 16.8, standard deviation 3.1), Spearman $\rho = 0.13$. **(b)** Wechsler Intelligence Scale for Children 5th Edition (WISC-V)[51] matrix reasoning score (mean 10, standard deviation 3, maximum 19), Spearman $\rho = -0.12$. Both correlations were significant at $p < 0.001$. Each point represents one participant in the ABCD study. Head motion was measured as framewise displacement (FD, in mm) averaged over resting-state scans. FD was log-transformed to improve fit. The best fit is shown as a dashed line.

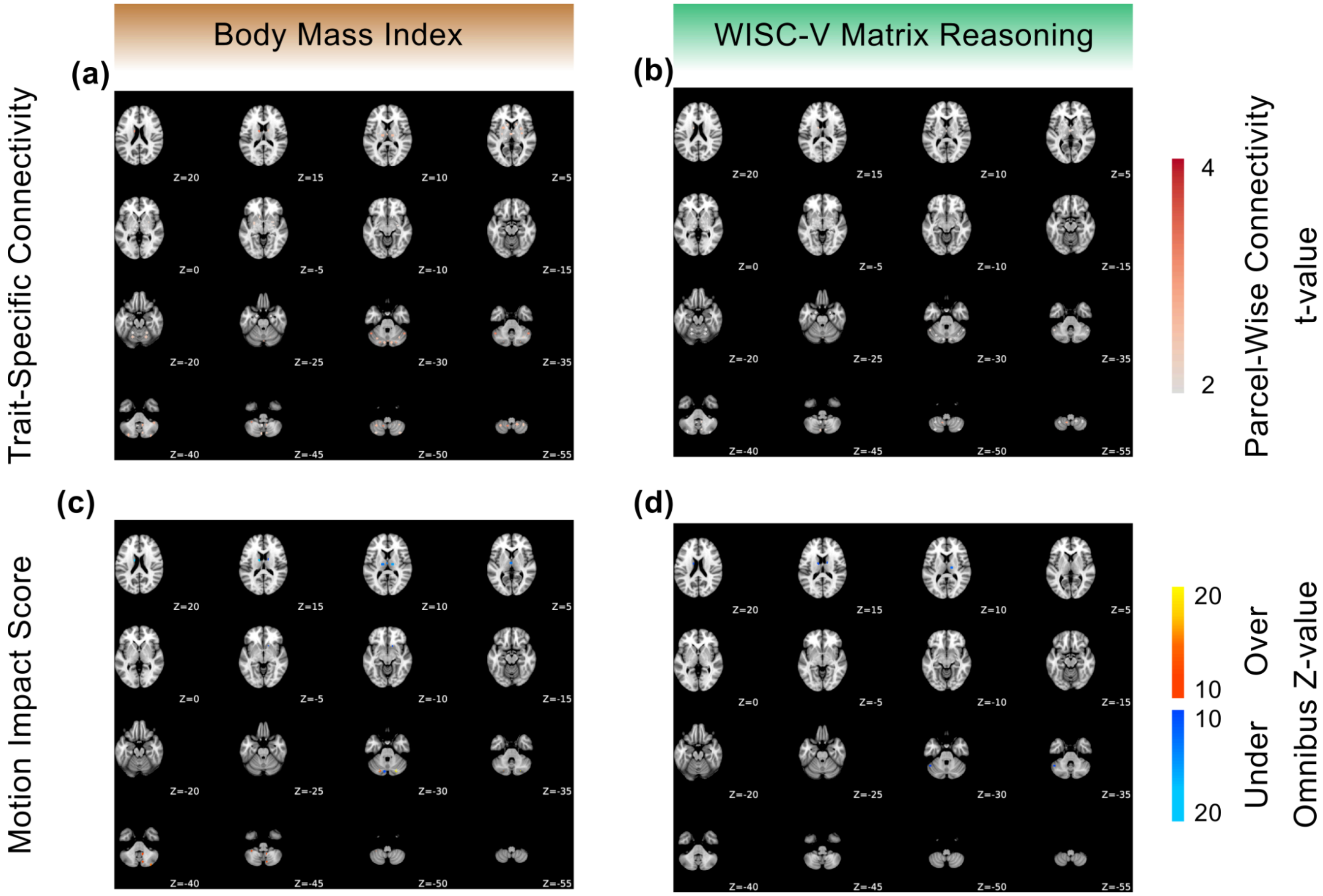

Supplementary Figure 3: Trait-specific impact of motion on functional connectivity (FC). This companion to Figure 4 renders results on the 61 Seitzman [66] subcortical regions of interest.**Top:** Parcel-wise FC, computed as the root mean square (RMS) of connectivity values for each parcel/node in the trait-FC effect matrix for**(a)** body mass index (BMI) and **(b)** WISC-V matrix reasoning score. **Bottom:** Motion impact score for **(c)** BMI and **(d)** WISC-V. Motion overestimation scores are labeled “Over” in orange and motion underestimation scores are labeled “Under” in blue.

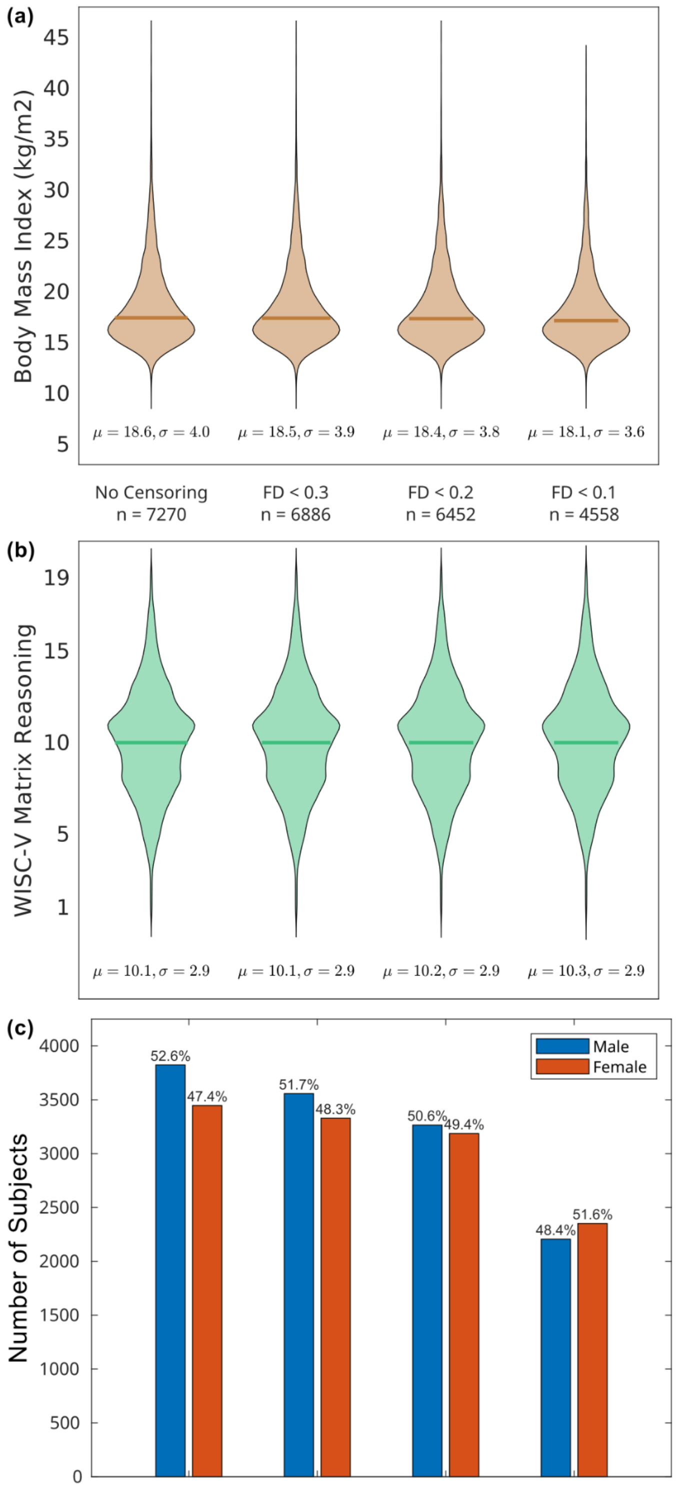

Supplementary Figure 4: Sampling bias at different levels of framewise censoring for selected variables. Sample distributions are shown for **(a)** body mass index (BMI), **(b)** WISC-V matrix reasoning score, and **(c)** sex assigned at birth. Mean (solid line) and standard deviation (SD) for (a) BMI and (b) matrix reasoning were similar across all levels of motion censoring, but the proportion of boys to girls shifted significantly from 52.6% without motion censoring to 51.6% with very stringent censoring at framewise displacement (FD) < 0.1 mm.

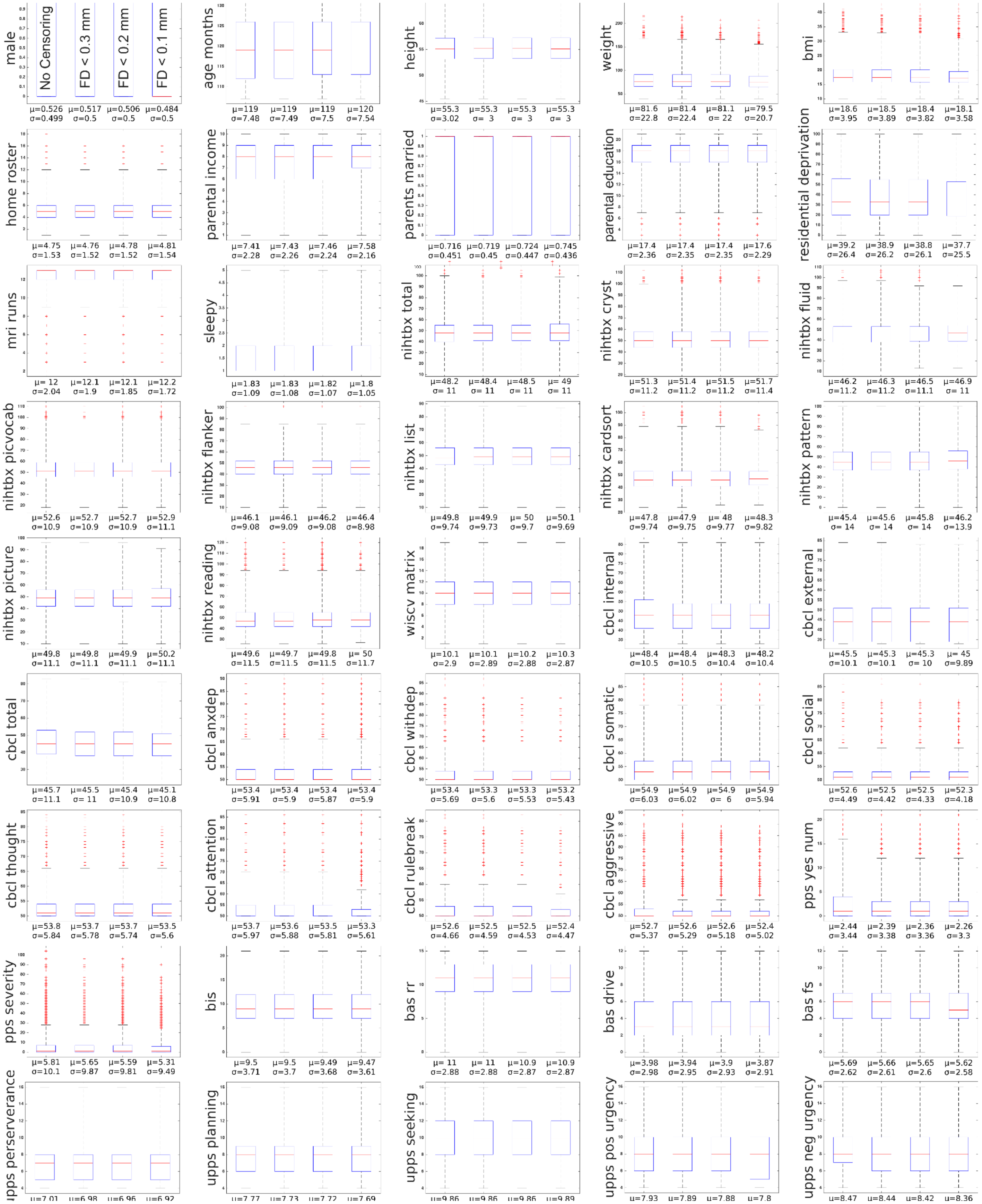

Supplementary Figure 5: Sampling bias at different levels of framewise censoring. Box and whisker plots are shown for all 45 traits examined in the ABCD study at different levels of motion censoring. From left to right, no censoring, FD < 0.3 mm, FD < 0.2 mm, and FD < 0.1 mm.

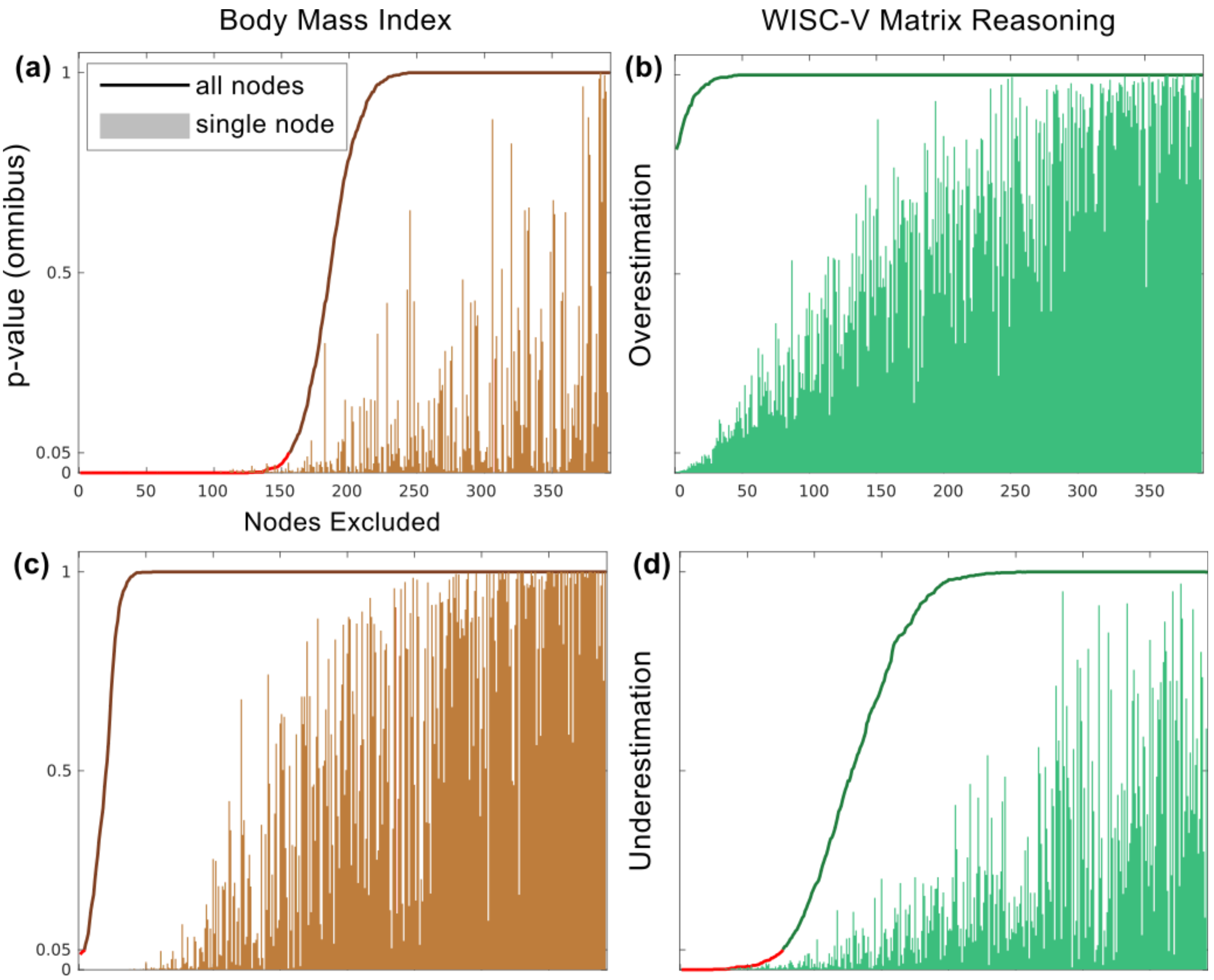

Supplementary Figure 6: Number of nodes contributing to significance of motion impact. Top: Nodes driving motion overestimation score for **(a)** body mass index (BMI) and **(b)** WISC-V matrix reasoning score. **Bottom:** Nodes driving motion underestimation score for **(c)** BMI and **(d)** matrix reasoning. Reading each plot from left to right, the node with the highest motion score (lowest omnibus p-value) was iteratively excluded, and the omnibus p-value for edges across all remaining nodes is plotted as a solid line. The line is red for $p < 0.05$ (significant motion associated connectivity) and black for $p > 0.05$ (not significant). The omnibus p-value for each single node excluded is shown in the background.

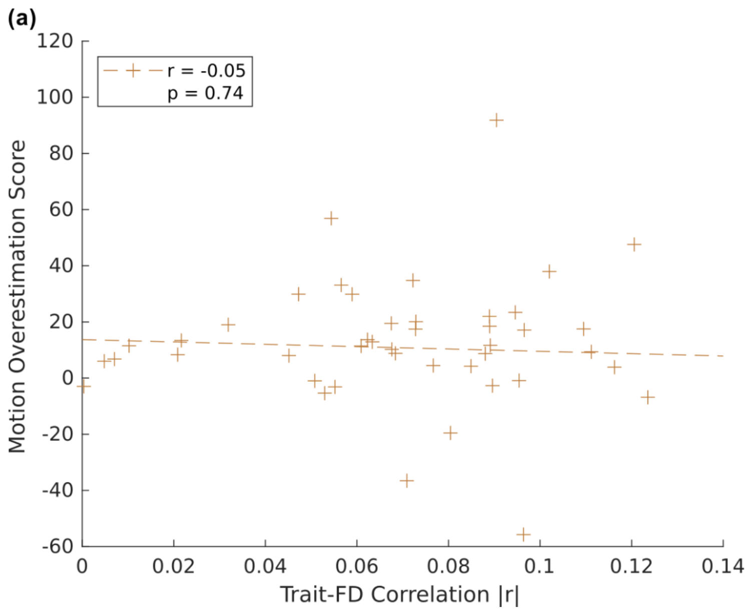

Supplementary Figure 7: Correlation between SHAMAN motion impact score and in-scanner head motion. (a) Motion overestimation and **(b)** motion underestimation scores (omnibus Stouffer's Z-score) for selected variables from the ABCD study are plotted vs the correlation (absolute value) of those variables with average participant head motion (framewise displacement, FD). Results reflect fMRI data after motion-reduction with ABCD-BIDS and without additional motion censoring. See Supplementary Data 1.

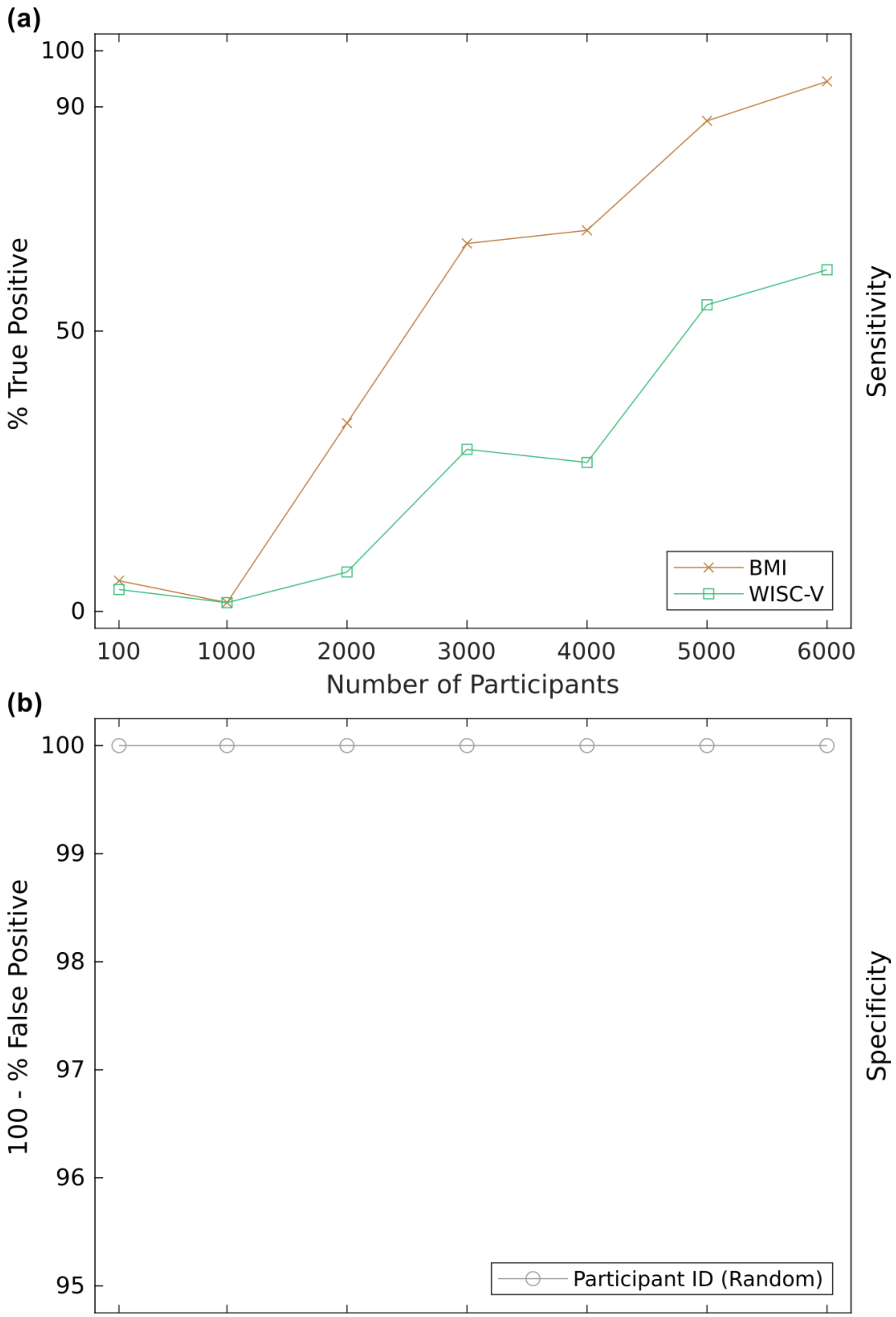

Supplementary Figure 8: Statistical Power of SHAMAN Method. (a) True positive detection rate (sensitivity, power) of SHAMAN computed using bootstrapped subsamples of different sizes (numbers of participants) with body mass index as an exemplar variable for significant motion overestimation score and WISC-V matrix reasoning score as an exemplar for motion underestimation score. **(b)** Specificity of motion impact score using a random variable generated from the unique participant identifier. All data use 128 bootstraps and 128 permutations at each sample size.

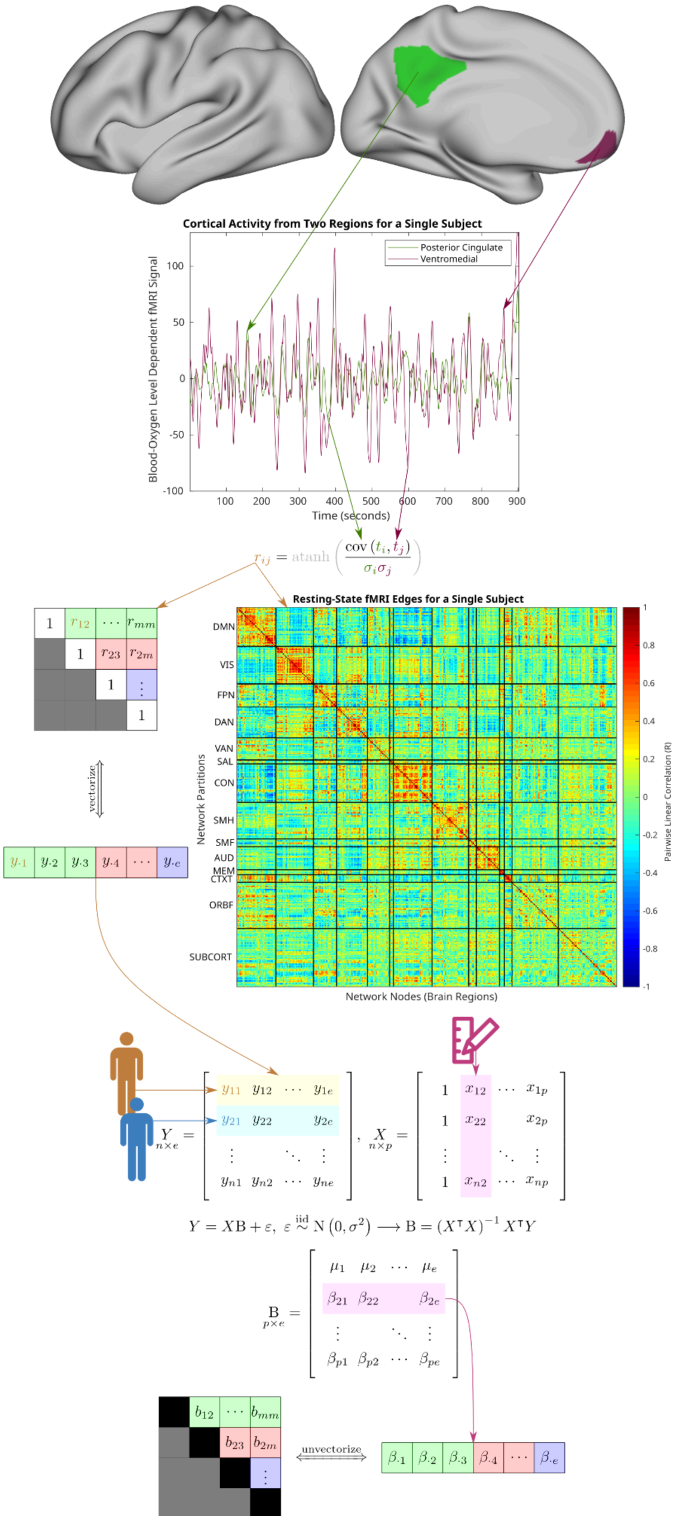

Supplementary Figure 9: Conventional regression model for resting-state functional connectivity.

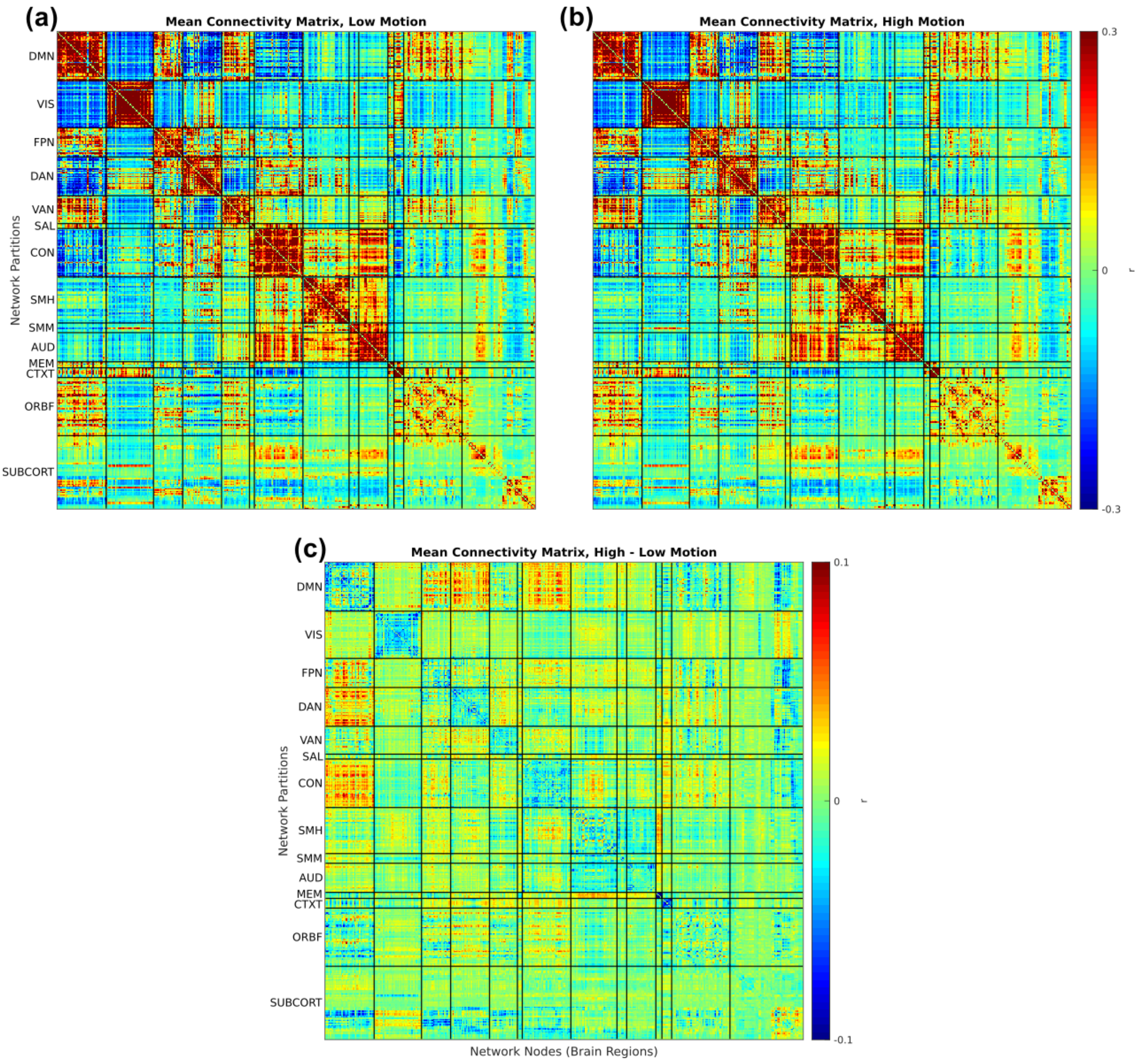

Supplementary Figure 10: Mean (average) connectivity matrix computed from the low-motion **(a)** and high-motion **(b)** halves of each participant's resting-state fMRI data. The difference (rescaled to show visual detail) is shown in **(c)**. Figures are generated from data after denoising with ABCD-BIDS but with*out* motion censoring.

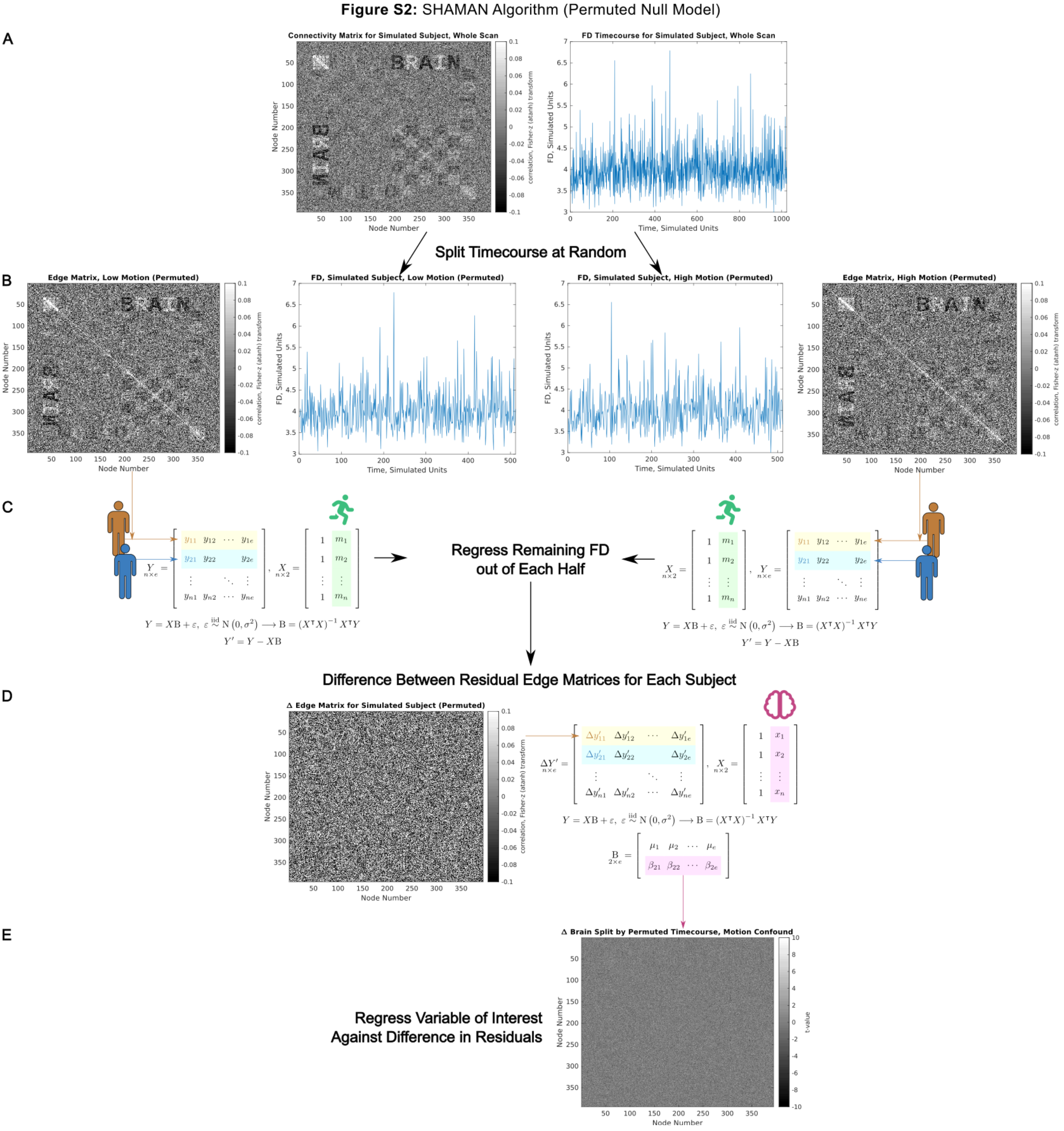

Supplementary Figure 11: Null model permutation scheme for SHAMAN algorithm. In **(B)** the data is split in half at random, without respect to the FD timeseries. The rest of the algorithm proceeds as before. The algorithm is repeated many times for each random split (permutation) to generate a null distribution for the motion-associated connectivity matrix in **(E)**.

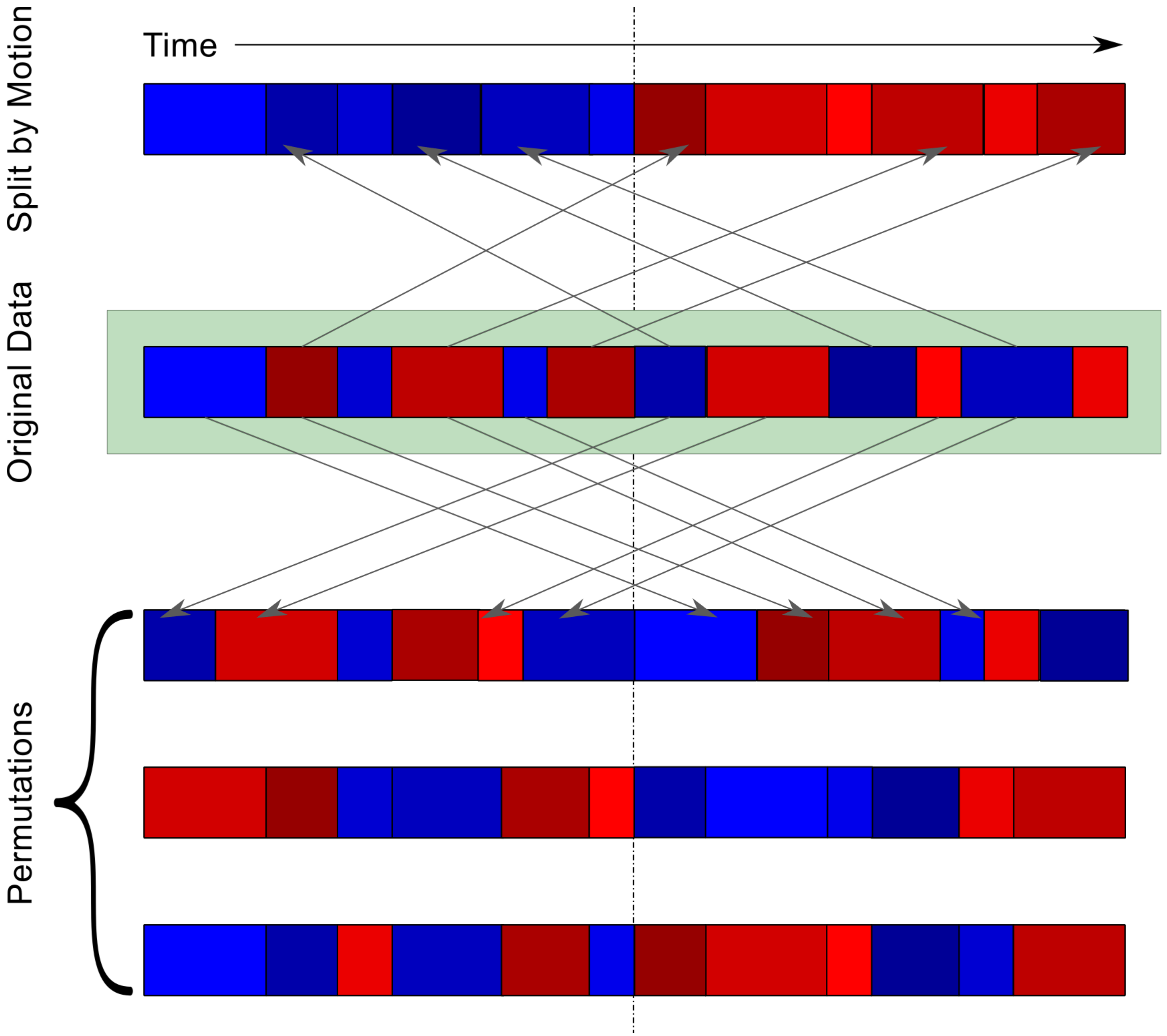

Supplementary Figure 12: Motion blocks permutation method. A single participant's fMRI timeseries data is visualized as a rectangle with time along the horizontal axis. A dashed vertical line represents division of the data into two halves. Data with associated motion (e.g. framewise displacement, FD) below the median is labeled blue, and data with motion above the median is labeled red. Consecutive timepoints labeled the same color form temporally consecutive "blocks" of data. Different shades of blue and red are used to highlight the boundaries of different motion-derived blocks. When split by motion **(top)**, all of the low-motion blocks and all of the high-motion blocks are grouped together in the same split half of the data. During permutation **(bottom)**, blocks are sorted randomly into each split half. Preserving temporally consecutive timepoints within blocks during permutation ensures the same amount of temporal autocorrelation in permuted and un-permuted divisions of the data.

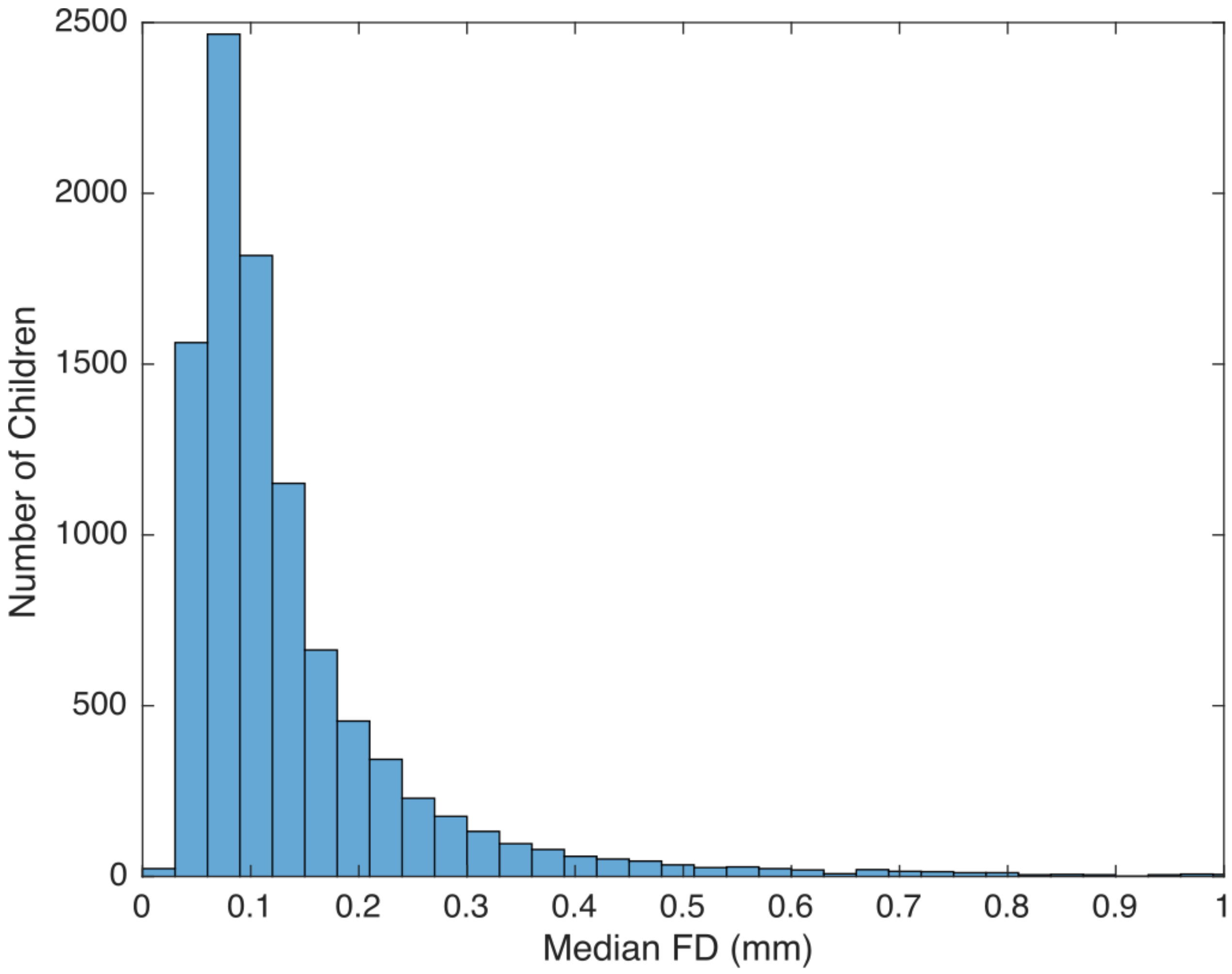

Supplementary Figure 13: Distribution of median FD. Data are shown for n = 9,625 children in the ABCD study with at least 8 minutes of rs-fMRI data before motion censoring. The histogram is truncated at 1 mm for readability; the maximum median FD was 2.9 mm.

Simulation

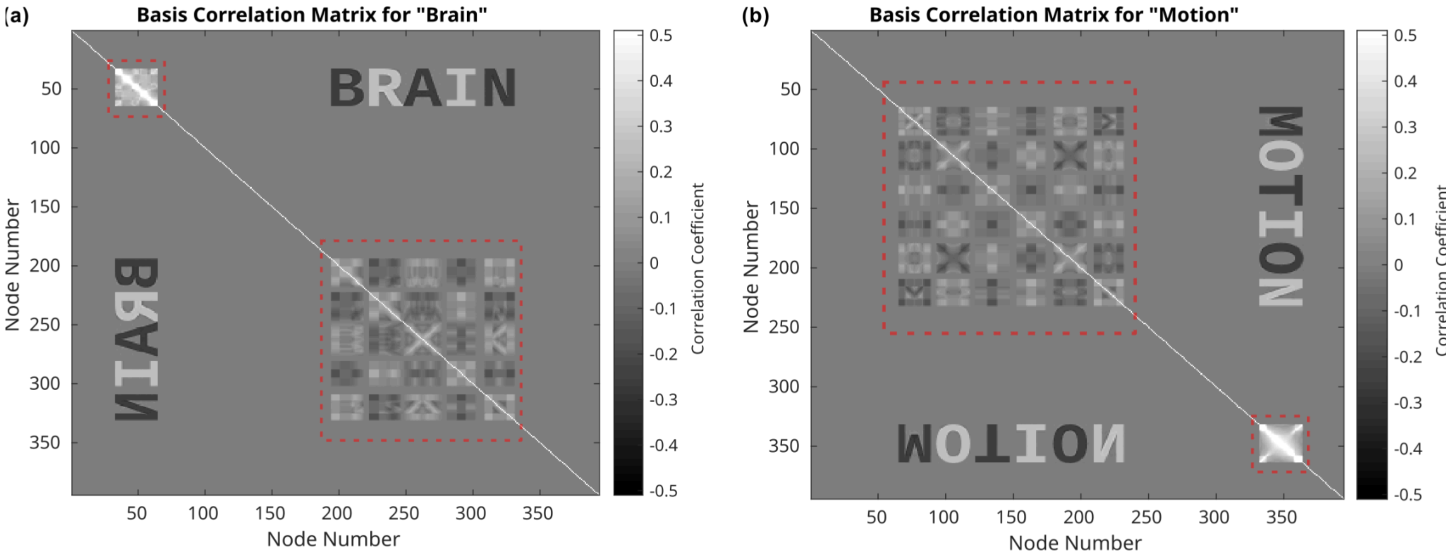

Supplementary Figure 14: Basis correlation matrices. (a) “Brain” signal. **(b)** “Motion” artifact. The dimensions of each matrix are 394 x 394 nodes. The portions of the matrices outlined in the red dashed boxes were algorithmically added to make each matrix positive semi-definite.

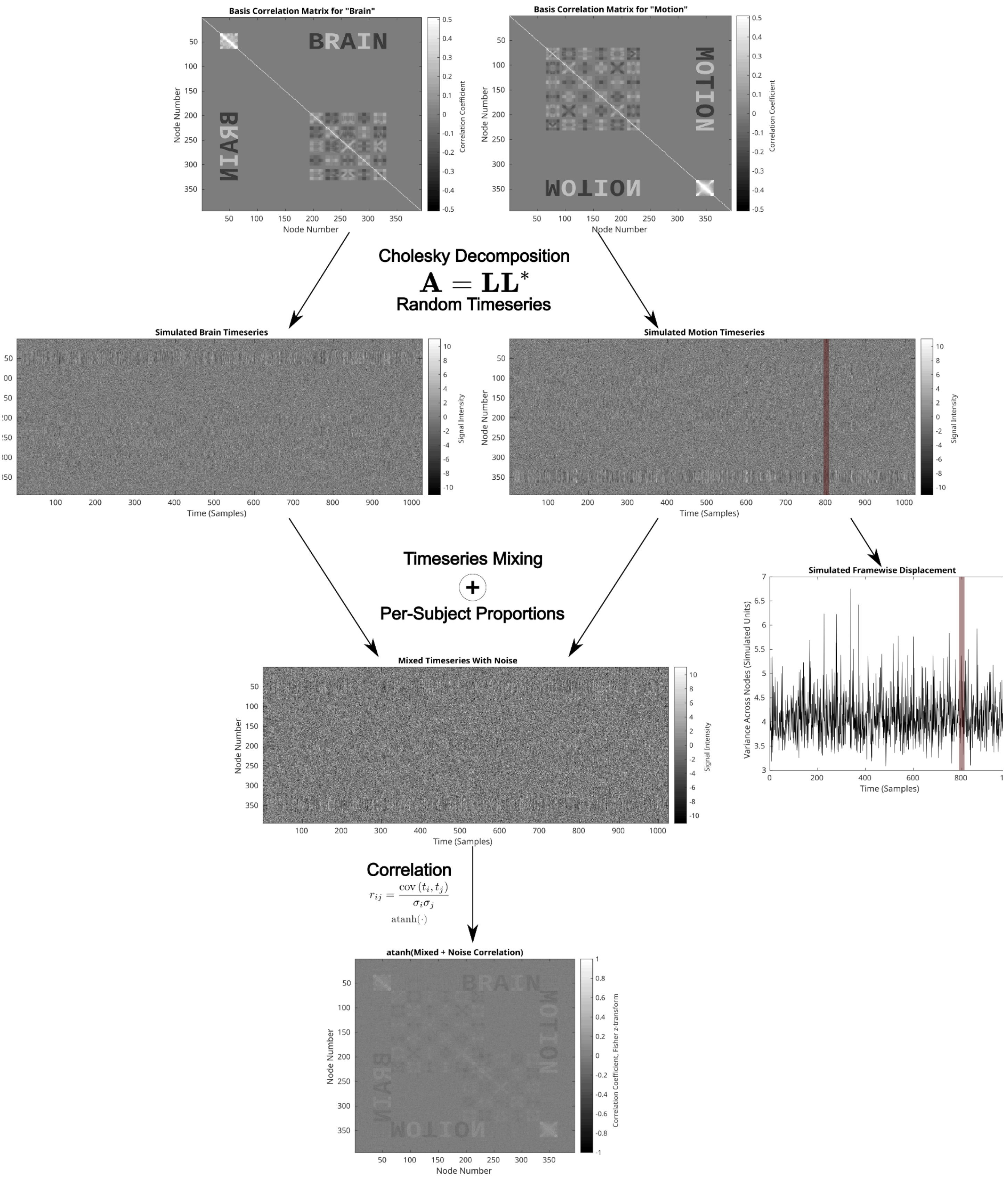

Supplementary Figure 15: Method for generating simulated fMRI timeseries and connectivity matrices. 394 x 394 basis correlation matrices for “brain” and “motion” were Cholesky-decomposed to generate random 394 x 1,024 timeseries for each of 1,024 participants. The simulated FD (framewise displacement) timeseries was computed from the across-node variance of the motion timeseries at each point in time.

Simulation Results

Simulation results are summarized in Supplementary Figure 16. First, as a negative control, we simulated FD timeseries but did not add any motion into the simulated fMRI data. This simulation depicts a scenario in which head motion occurred during scanning, but motion denoising algorithms were able to perfectly remove it from the data. No motion was seen in the mean connectivity matrix nor the brain-specific connectivity matrix from regression, nor did SHAMAN detect any trait-specific motion impact.

As a second negative control, we simulated cases where motion *was* mixed into the data in equal proportion to brain signal, and across-participant motion was linearly separable from brain signal. This linear separability occurred for simulations where the proportions of brain and motion signal across participants were uncorrelated (i.e. trait/variable is not correlated with motion at all), and also for simulations where the proportions of brain and motion signal in the design matrix were linearly related to the true proportions of brain and motion signal mixed into the data (i.e. FD is a highly-accurate linear function of motion-induced fMRI signal). For these simulations we did observe motion in the mean connectivity matrix (Supplementary Figure 16a) but there was no motion in the trait-specific connectivity matrix (Supplementary Figure 16c). SHAMAN correctly did not detect any motion impact (Supplementary Figure 16e).

As a positive control, we simulated cases where motion was mixed into the data in equal proportion to brain signal, but the FD timeseries was not a linear function of the proportion of mixed motion signal. In Supplementary Figure 16, second column, we achieved this by drawing the motion-signal timeseries, x, from a random normal distribution correlated with the simulated brain signal $r = 0.5$. We used x to compute FD, but then transformed the motion-signal timeseries by $f(x) = 1 + x^2$ before mixing (i.e. FD is related to motion but not a pure linear function of it). As expected, we observed motion in the mean connectivity matrix (Supplementary Figure 16b). We also observed residual motion artifact in the brain/trait-specific connectivity matrix from regression (Supplementary Figure 16d), even when modeling mean FD as a covariate in regression. SHAMAN correctly detected the presence of this trait-specific motion impact (Supplementary Figure 16f).

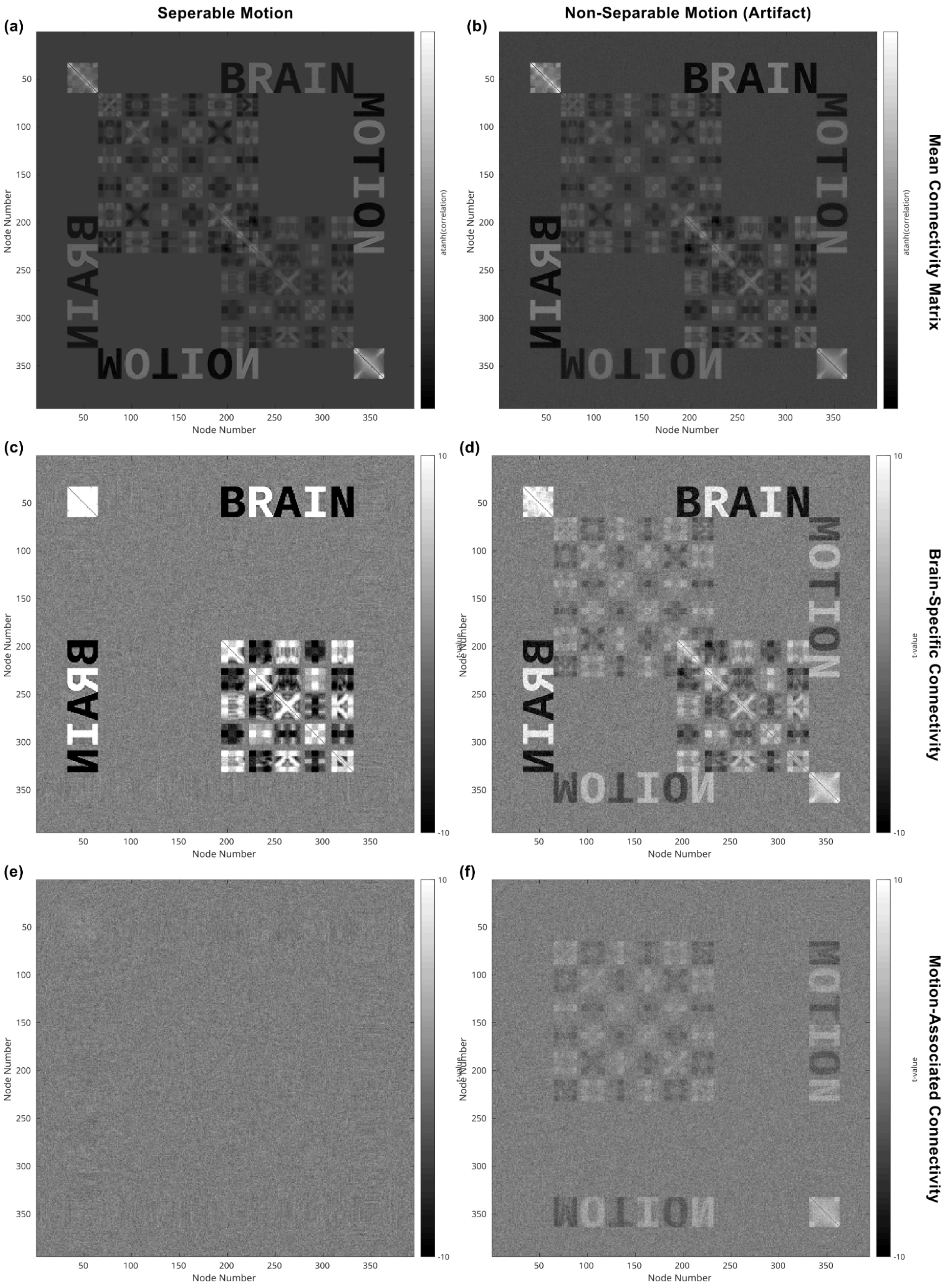

Supplementary Figure 16: Simulation results when motion is linearly separable (left) and when motion is not linearly separable (right). In both cases **(a, b)** motion is present in the mean connectivity matrix. The linear regression model for “brain” with motion (participants’ mean FD) as a covariate successfully removes the motion in **(c)** but not in **(d)**. SHAMAN does not detect motion-associated connectivity in **(e)** but correctly detects the artifact in **(f)** ($p < 0.001$).

Variance Correction

Proper timeseries mixing requires an additional step of variance correction to account for assumptions in the conventional model for resting-state fMRI regression. This model, diagrammed in Supplementary Figure 9, assumes that connectivity matrices (derived from correlation matrices) are linearly superimposable for the purposes of linear regression. Correlation is computed by taking the covariance of two timeseries and dividing by the product of the standard deviations of the timeseries. Thus, regardless of the total amount of variation in each timeseries, the correlation is constrained to vary between -1 and 1. Therefore, in order to make correlation matrices (or their atanh-transformed connectivity matrices) linearly superimposable, we simulated data with the same total variance across participants.

Furthermore, we assume that (after any time-series/autoregressive filtering during pre-processing) computing correlation does not depend on the temporal ordering of the fMRI timeseries. This assumption allows us to "censor" high-motion frames from a timeseries, or to concatenate timeseries from different scans together. Therefore, in addition to generating data with the same total variance across participants, we simulated data with the same total variance across time.

The process for mixing the simulated timeseries to satisfy these assumptions is diagrammed in Supplementary Figure 17. First the brain and motion timeseries were added together to generate a provisionally-mixed timeseries. Then the target variance to use across all participants was computed by summing the columns of the target design matrix and finding the row with the maximum value corresponding to the participant with the highest, or "maximum" variance. A random variance-correction timeseries was generated for each participant such that, when added to the provisionally-mixed timeseries, the resultant variance-corrected timeseries had constant across-node variance over time equal to the maximum variance.

Finally, an equal amount of normally-distributed noise was added to the variance-corrected timeseries for each participant to obtain the final, properly-mixed timeseries used to compute each participant's correlation and connectivity matrices. This additional noise models unstructured noise from the fMRI scan (e.g. thermal noise), and also allows for the across-node variance of the mixed timeseries to vary with time while still satisfying the assumptions of the linear model.

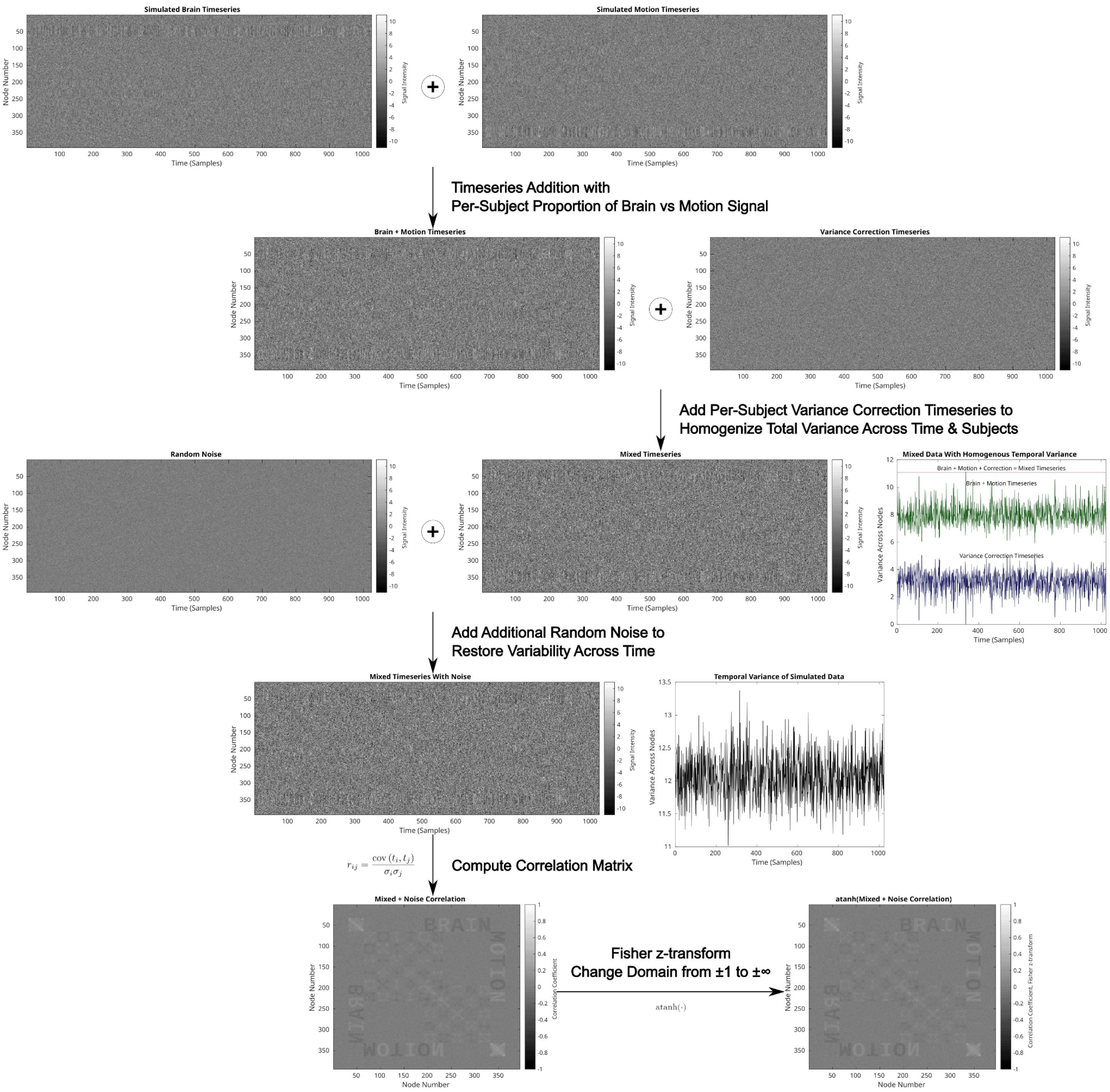

Supplementary Figure 17: Method for proper timeseries mixing of brain and motion signals in controlled proportions. A provisional mixing of the brain and motion timeseries is obtained by addition in the time domain. A random variance-correction timeseries is then added such that, at each time point, the across-node variance of the variance-corrected timeseries is equal to the maximum variance of any participant. Finally, an equal amount of normally distributed noise is added to the variance-corrected timeseries to simulate thermal noise and to allow for the across-node variance of the final, properly-mixed timeseries to vary with time. The mixed timeseries are used to generate connectivity matrices for each participant as in Supplementary Figure 15.

Data Comparison

Rationale

Innumerable methods exist for quantifying and mitigating head motion artifact within many large neuroimaging data sets. DVARS, an alternative to framewise displacement (FD) for quantifying in-scanner head motion, was first proposed by Smyer et al[72] and later popularized by Power[14] and Afyouni & Nichols[73]. More recently, Pham et al[41] demonstrated a method for selecting an optimal DVARS motion censoring threshold. DVARS is an acronym for, "D referring to temporal derivative of timecourses, VARS referring to RMS variance over voxels."[14] It is calculated as the root mean square difference in voxel intensity (within a brain mask) between one fMRI timepoint/volume and the next. A critical conceptual difference between FD and DVARS is that FD measures motion specifically, being itself derived from the six rigid-body motion co-registration parameters, whereas DVARS is sensitive to any change in fMRI signal intensity, whether due to motion artifact or another source.

In this supplementary analysis we apply SHAMAN to data processed with different exemplar methods and from different data sets. Specifically, we apply SHAMAN to ABCD data using DVARS in place of FD, and to Human Connectome Project (HCP) data, [49,50] which is processed using ICA-FIX [7,16] using both FD and DVARS. The purpose of this supplement is not to exhaustively catalog all possible methodological variations, nor to make specific prescriptions for data processing. Rather, we show that the general trends seen in our main analysis are replicated in different data processed in different ways.

Methods

HCP Data Acquisition and Processing

The Human Connectome Project (HCP) includes fMRI data from 1200 young, healthy adult volunteers [49,50]. Resting-state fMRI data was acquired at 3T using gradient-echo EPI with TR = 720 ms, slice thickness 2.0 mm, and 72 slices for 2.0 mm isotropic voxels. There were 4 resting state runs with 1200 frames per run [74]. We used data after minimal processing [38] and ICA-FIX [7,16]. Part of our rationale for analyzing HCP data was to test SHAMAN on data processed with ICA-FIX, which automatically removes motion artifact using independent component analysis. As with the ABCD data, at each level of motion censoring, we excluded participants with fewer than 600 fMRI frames after censoring. This resulted in n = 1017 (out of 1200 participants recruited) with usable resting-state fMRI data before censoring. For computational expediency, we only ran 128 permutations of SHAMAN for each motion measure and censoring threshold in HCP data.

Calculation of FD and DVARS in HCP

FD and DVARS were computed using the methods described by Power et al [14] with the following modifications from ABCD. FD was computed without correction for respiratory motion artifact, which is part of the ABCD-BIDS pipeline. The FD censoring thresholds were the same as those used in the ABCD data: 0.3, 0.2, and 0.1 mm. DVARS was computed using the normalized method of Afyouni & Nichols [73] as implemented by the Nipype software. We selected a threshold of 200 for consistency with the ABCD data.

Selection of Traits in HCP

The following traits of interest in HCP were selected to parallel the traits selected in ABCD. The meanings of variable names can be found in the HCP data dictionary: https://wiki.humanconnectome.org/display/PublicData/HCP-YA+Data+Dictionary-+Updated+for+the+1200+Subject+Release

Demographic:

SSAGA_Income, SSAGA_Educ

Physical:

Age_in_Yrs, Height, Weight, BMI, Handedness, Hematocrit_1, ThyroidHormone, HbA1C, Dexterity_AgeAdj, Strength_AgeAdj, Noise_Comp, Odor_AgeAdj, Taste_AgeAdj

Personality:

PSQI_Score, DDisc_AUC_200, DDisc_AUC_40K, ER40_CR, ER40_CRT, AngAffect_Unadj, AngHostil_Unadj, AngAggr_Unadj, FearAffect_Unadj, FearSomat_Unadj, Sadness_Unadj, LifeSatisf_Unadj, MeanPurp_Unadj, PosAffect_Unadj, Friendship_Unadj, Loneliness_Unadj, PercHostil_Unadj, PercReject_Unadj, EmotSupp_Unadj, InstruSupp_Unadj, PercStress_Unadj, SelfEff_Unadj, NEOFAC_A, NEOFAC_O, NEOFAC_C, NEOFAC_E, ASR_Anxd_Pct, ASR_Witd_T, ASR_Soma_T, ASR_Thot_T, ASR_Attn_T, ASR_Aggr_T, ASR_Rule_T, ASR_Intr_T, ASR_Intn_T, ASR_Extn_T, ASR_Totp_T, DSM_Depr_T, DSM_Anxi_T, DSM_Somp_T, DSM_Avoid_T, DSM_Adh_T, DSM_Antis_T

Cognition:

MMSE_Score, PicSeq_AgeAdj, CardSort_AgeAdj, Flanker_AgeAdj, PMAT24_A_CR, PMAT24_A_RTCR, ReadEng_AgeAdj, PicVocab_AgeAdj, ProcSpeed_AgeAdj, VSPLOT_TC, VSPLOT_CRTE, IWRD_TOT, IWRD_RTC, ListSort_AgeAdj, CogFluidComp_AgeAdj, CogTotalComp_AgeAdj, CogCrystalComp_AgeAdj

Calculation of FD and DVARS

FD was computed using the method described by Power et al [14]. In the ABCD data (but not the HCP data), the ABCD-BIDS pipeline [25,37] computed FD with correction for respiratory motion artifact. FD censoring thresholds of 0.3, 0.2, and 0.1 mm were selected for both studies. DVARS was computed using the method and software of Afyouni & Nichols [73]; MATLAB code is available at: https://github.com/asoroosh/DVARS. A manual and an automatic DVARS censoring threshold were selected. A Manual DVARS threshold of 200 excluded 1,058 participants (retention of 6,212 participants) from the ABCD data; no participants were excluded from HCP by this threshold. An second, automatic DVARS threshold was applied using the principled cutoff technique of Afyouni & Nichols [73] by excluding frames with a DVARS p-value < 0.05 (including frames with $p > 0.05$). The DVARS $p < 0.05$ cutoff excluded 541 participants from ABCD (retention of 6,729 participants) and did not exclude any participants from HCP.

Results

Head Motion and Motion Impact Scores Were Lower in HCP

The ABCD and HCP data sets differed substantially in terms of sample size (ABCD n = 7270, HCP n = 1017), age (ABCD 9.9 ± 0.6 years, HCP 28.7 ± 3.7 years), and amount of in-scanner head motion. Mean FD was greater in ABCD, 0.29 ± 0.35 mm, compared to HCP, 0.16 ± 0.06 mm, see Supplementary Figure 18. The distribution of mean FD was heavier-tailed in ABCD. The largest mean FD in the ABCD data was 6.0 mm.

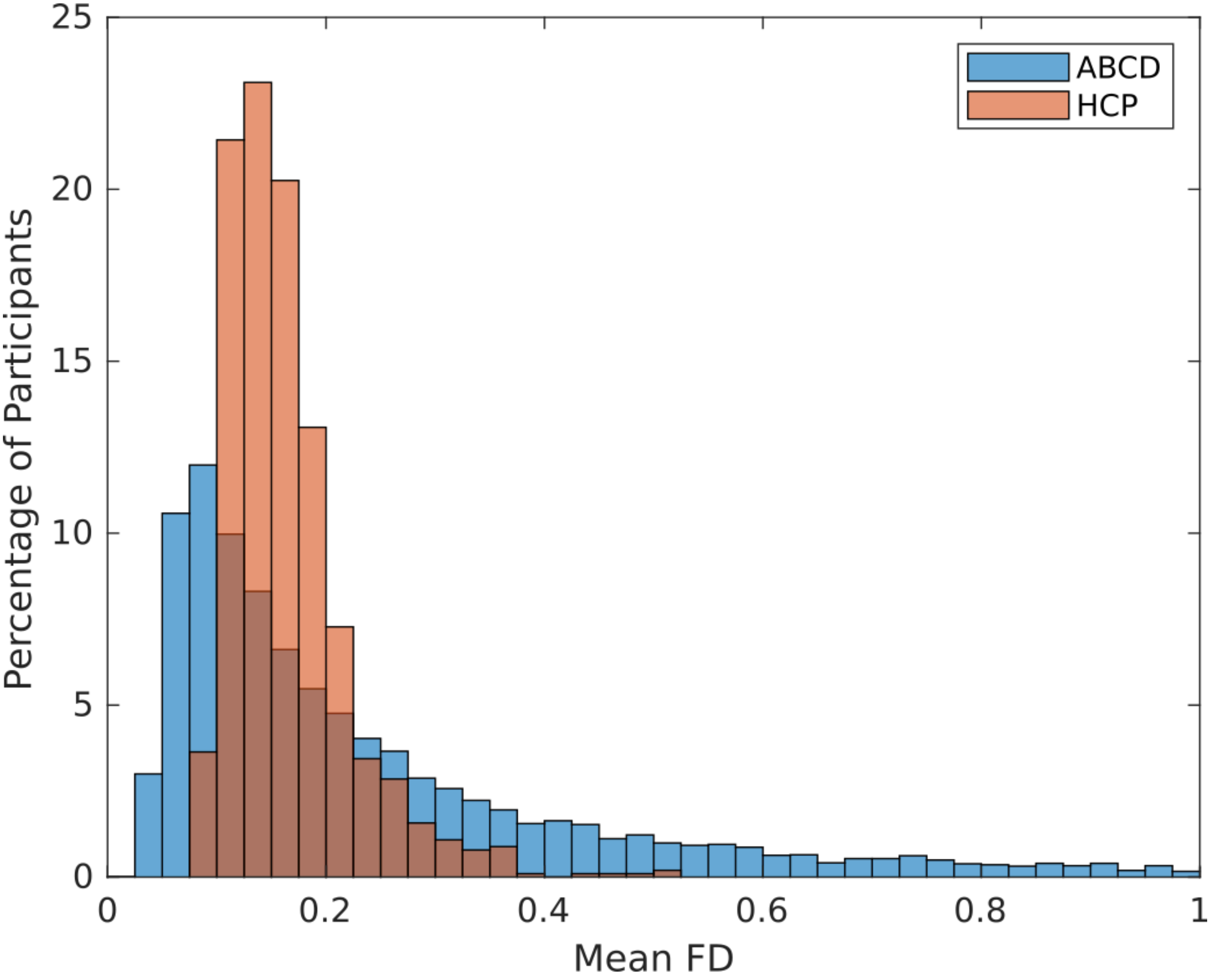

Supplementary Figure 18: In-scanner head motion in ABCD and HCP. Head motion was quantified by mean framewise displacement (in mm). The histogram for ABCD is truncated at FD = 1.0 mm. The largest mean FD in the ABCD data is 6.0 mm. Data are shown without motion censoring.

WIthout motion censoring, 13% (10/76) of traits in HCP had a significant (omnibus $p < 0.05$) motion impact score (over- or under-estimation of the trait-FC effect) compared to 53% (24/45) traits in ABCD. 12% (9/76) of traits in HCP had a significant motion overestimation score and 14% (11/76) had a significant motion underestimation score. Our selection of personality traits in HCP covered a wider variety of tests than the personality variables we selected from ABCD. Personality traits were the most numerous (16%, 7/43) to have significant motion impact scores in HCP (either over- or under-estimation), but as in ABCD, physical traits in HCP were still the most likely to have a significant motion impact score (23%, 3/13).

Motion Censoring Reduced Motion Impact Score in HCP

As in ABCD, motion censoring reduced motion impact scores in HCP. Only two traits, age and NIH Toolbox anger-aggression subscore, had significant motion overestimation scores at FD < 0.3 mm. Only NIH Toolbox card sorting subscore had significant motion overestimation at FD < 0.1 mm. While the motion overestimation score for age remained highly significant at FD < 0.2 mm ($p < 0.001$), the motion impact scores of the toolbox measures may have been spuriously significant due to the low number of permutations performed ($p = 0.047$). Motion censoring was more effective at reducing motion underestimation scores in HCP. Besides age, no traits in HCP had a significant motion underestimation score in HCP after censoring at FD < 0.2 mm. See Supplementary Figure 19 and Supplementary Data 6-8.

Unlike ABCD, in which motion censoring at FD < 0.2 mm excluded 818 participants due to insufficient remaining data, motion censoring HCP at FD < 0.2 mm did not result in the exclusion of any participants. Motion censoring HCP at FD < 0.1 mm resulted in the exclusion of 89 participants (8.8%), with 928 participants remaining.

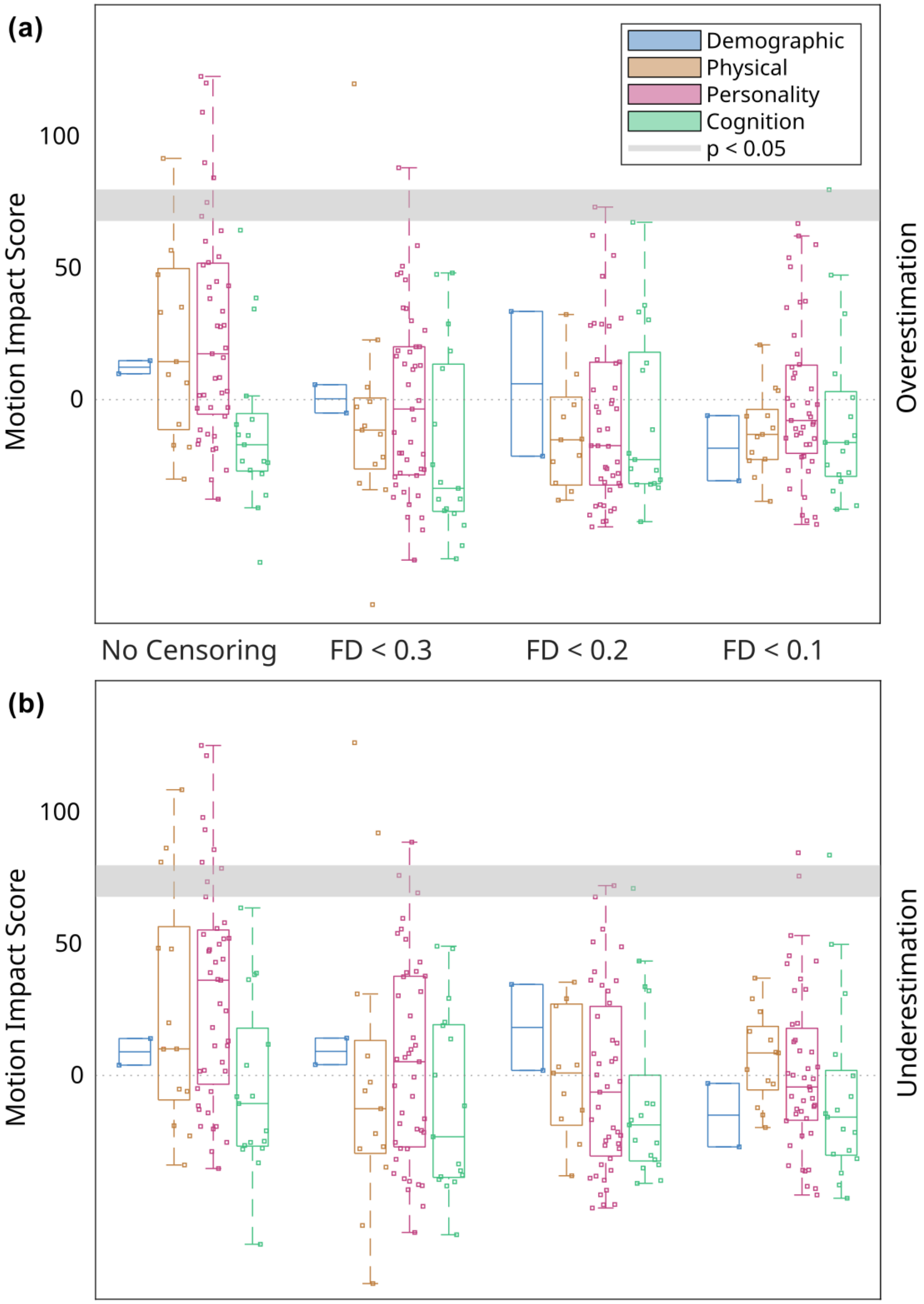

Supplementary Figure 19: Effects of frame censoring on motion impact score in HCP data using FD. Motion impact score (omnibus Stouffer's Z, higher = more motion) for traits of a given category at different levels of motion censoring. The cutoff score for significance at $p < 0.05$ is different for each trait. The range of significance thresholds is indicated by a gray band. **(a)** Motion overestimation score. **(b)** Motion underestimation score.

Motion Impact Scores Were Similar for FD and DVARS

Motion impact scores, and the effect of motion censoring, were comparable for FD and DVARS. There were 7270 participants with usable fMRI in ABCD and 1017 participants in HCP prior to censoring. Frame censoring at FD < 0.3 mm retained 6886 participants in ABCD and 1017 in HCP, 0.2 mm left 6452 in ABCD and 1017 in HCP, and 0.1 mm left 4558 in ABCD and 928 in HCP. Frame censoring at DVARS < 200 retained 6212 participants in ABCD and 1017 in HCP. Frame censoring at DVARS p > 0.05 retained 6,729 participants in ABCD and 1017 in HCP. Using DVARS in ABCD, 82% (37/45) of traits had a significant motion overestimation score and 13% (6/45) had a significant motion underestimation score without motion censoring. After motion censoring at DVARS p > 0.05, 16% (7/45) of traits had a significant motion overestimation score and 11% (5/45) of traits had a significant motion underestimation score. Using DVARS in HCP, 2/45 traits had significant motion overestimation score before censoring and no traits had significant overestimation score after censoring at DVARS p > 0.05 with no exclusion of participants. See Supplementary Figures 19-20 and Supplementary Data 9-12.

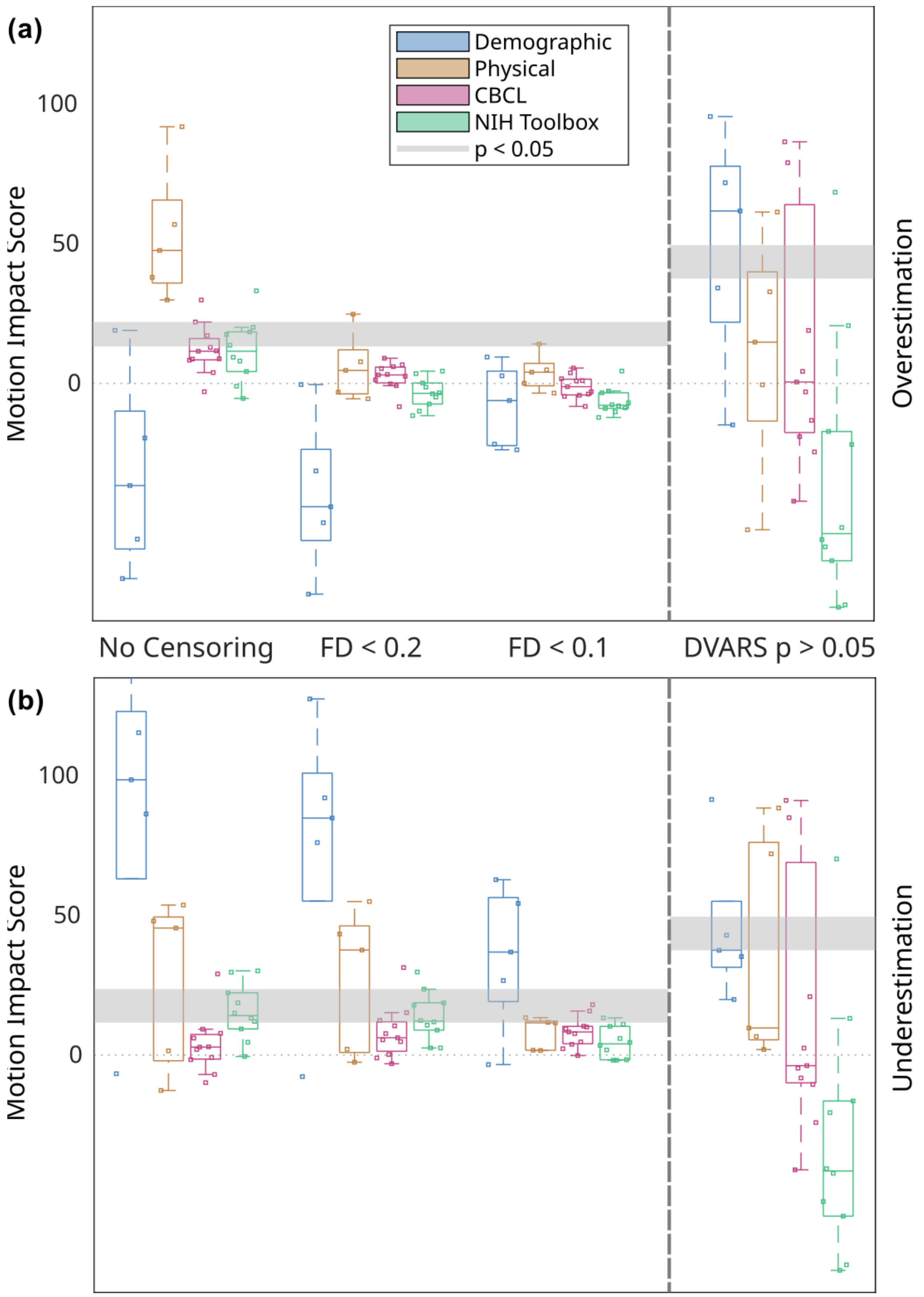

Supplementary Figure 20: Effects of frame censoring on motion impact score in ABCD data using FD vs DVARS. Motion impact score (omnibus Stouffer's Z, higher = more motion) for traits of a given category at different levels of motion censoring. The cutoff score for significance at $p < 0.05$ is different for each trait. The range of significance thresholds is indicated by a gray band. **(a)** Motion overestimation score. **(b)** Motion underestimation score.

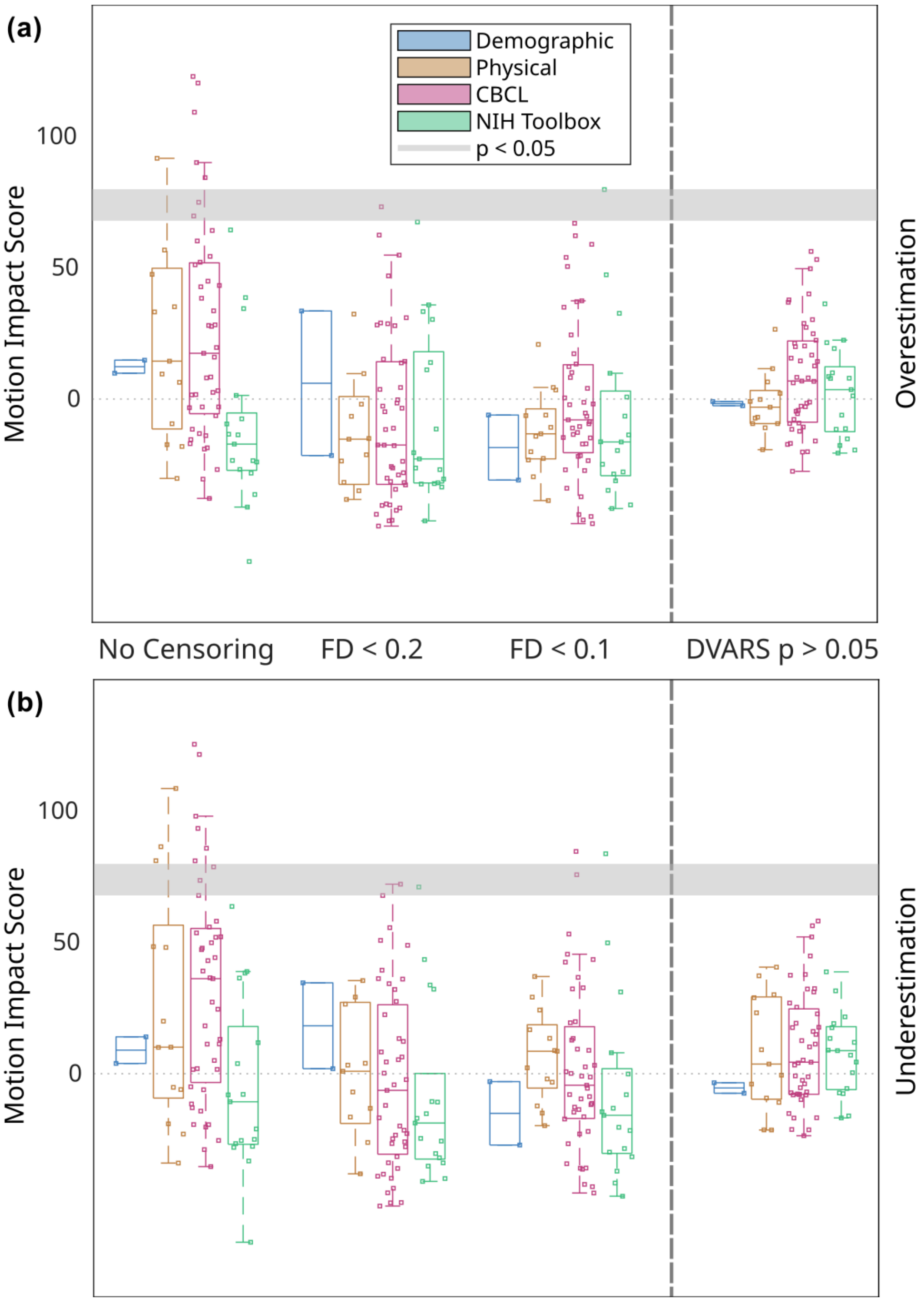

Supplementary Figure 21: Effects of frame censoring on motion impact score in HCP data using FD vs DVARS. Motion impact score (omnibus Stouffer's Z, higher = more motion) for traits of a given category at different levels of motion censoring. The cutoff score for significance at $p < 0.05$ is different for each trait. The range of significance thresholds is indicated by a gray band. **(a)** Motion overestimation score. **(b)** Motion underestimation score.

Discussion

Our supplementary analyses, using imaging data from the Human Connectome Project (HCP) in addition to ABCD and using DVARS as an alternative to framewise displacement for quantifying head motion, demonstrate a broadly consistent pattern of results applying SHAMAN to compute motion impact score. One limitation of our analysis was intrinsic differences in data processing, sample size, sample population, and amount of head motion in ABCD and HCP. The HCP study was processed using ICA-FIX, it had fewer participants, the participants were adults, and the participants had considerably less in-scanner head motion than the children in ABCD, consistent with prior observations of children as a high-motion cohort [19,22,29]. This made it difficult to disentangle whether lower motion impact scores in HCP were due to superiority of ICA-FIX as a method for mitigating head motion artifact, limitations in SHAMAN's sensitivity at smaller sample sizes, or lower head motion in the adult cohort. Despite these differences, clear parallels emerged in both data sets. Multiple traits, especially physical traits such as BMI, had significant motion overestimation scores in both ABCD and HCP. Age had a greater motion overestimation score in HCP, consistent with the wider distribution of ages in the HCP cohort. Motion censoring using FD < 0.2 mm reduced to near-zero the number of traits with significant motion overestimation scores in both ABCD and HCP. (Remarkably, in HCP, this reduction was possible without the exclusion of any participants at FD < 0.2 mm.) Thus, data processed with ICA-FIX are not immune to residual motion, and it is reasonable to suspect that residual motion artifact is an issue in most resting-state fMRI data regardless of processing approach.

The supplementary analysis comparing FD to DVARS revealed advantages and disadvantages of each method for quantifying in-scanner head motion. DVARS is sensitive to sources of transient fMRI signal change due to any cause, not just head motion. Combining DVARS with automatic selection of an optimal censoring cutoff [73] gave greatest reduction in motion overestimation in HCP data, but it underperformed an FD cutoff of 0.2 mm in ABCD data. The difference may have been related to differences in processing method and motion characteristics between ABCD and HCP data.

References

1. Behzadi Y, Restom K, Liau J, Liu TT. A component based noise correction method (CompCor) for BOLD and perfusion based fMRI. *NeuroImage*. 2007;37(1):90-101. doi:10.1016/j.neuroimage.2007.04.042
2. Bolton TAW, Kebets V, Glerean E, et al. Agito ergo sum: Correlates of spatio-temporal motion characteristics during fMRI. *NeuroImage*. 2020;209:116433. doi:10.1016/j.neuroimage.2019.116433
3. Burgess GC, Kandala S, Nolan D, et al. Evaluation of Denoising Strategies to Address Motion-Correlated Artifacts in Resting-State Functional Magnetic Resonance Imaging Data from the Human Connectome Project. *Brain Connect*. 2016;6(9):669-680. doi:10.1089/brain.2016.0435
4. Ciric R, Wolf DH, Power JD, et al. Benchmarking of participant-level confound regression strategies for the control of motion artifact in studies of functional connectivity. *NeuroImage*. 2017;154:174-187. doi:10.1016/j.neuroimage.2017.03.020
5. Dosenbach NUF, Koller JM, Earl EA, et al. Real-time motion analytics during brain MRI improve data quality and reduce costs. *NeuroImage*. 2017;161:80-93. doi:10.1016/j.neuroimage.2017.08.025
6. Fair DA, Nigg JT, Iyer S, et al. Distinct neural signatures detected for ADHD subtypes after controlling for micro-movements in resting state functional connectivity MRI data. *Front Syst Neurosci*. 2013;6. doi:10.3389/fnsys.2012.00080
7. Griffanti L, Salimi-Khorshidi G, Beckmann CF, et al. ICA-based artefact removal and accelerated fMRI acquisition for improved resting state network imaging. *NeuroImage*. 2014;95:232-247. doi:10.1016/j.neuroimage.2014.03.034
8. Kundu P, Brenowitz ND, Voon V, et al. Integrated strategy for improving functional connectivity mapping using multiecho fMRI. *Proc Natl Acad Sci*. 2013;110(40):16187-16192. doi:10.1073/pnas.1301725110
9. Makowski C, Lepage M, Evans AC. Head motion: the dirty little secret of neuroimaging in psychiatry. *J Psychiatry Neurosci*. 2019;44(1):62-68. doi:10.1503/jpn.180022
10. Mowinckel AM, Espeseth T, Westlye LT. Network-specific effects of age and in-scanner subject motion: A resting-state fMRI study of 238 healthy adults. *NeuroImage*. 2012;63(3):1364-1373. doi:10.1016/j.neuroimage.2012.08.004
11. Muschelli J, Nebel MB, Caffo BS, Barber AD, Pekar JJ, Mostofsky SH. Reduction of motion-related artifacts in resting state fMRI using aCompCor. *NeuroImage*. 2014;96:22-35. doi:10.1016/j.neuroimage.2014.03.028
12. Nielsen AN, Greene DJ, Gratton C, Dosenbach NUF, Petersen SE, Schlaggar BL. Evaluating the Prediction of Brain Maturity From Functional Connectivity After Motion Artifact Denoising. *Cereb Cortex*. 2019;29(6):2455-2469. doi:10.1093/cercor/bhy117
13. Patel AX, Kundu P, Rubinov M, et al. A wavelet method for modeling and despiking motion artifacts from resting-state fMRI time series. *NeuroImage*. 2014;95:287-304. doi:10.1016/j.neuroimage.2014.03.012
14. Power JD, Barnes KA, Snyder AZ, Schlaggar BL, Petersen SE. Spurious but systematic correlations in functional connectivity MRI networks arise from subject motion. *NeuroImage*. 2012;59(3):2142-2154. doi:10.1016/j.neuroimage.2011.10.018
15. Pruim RHR, Mennes M, van Rooij D, Llera A, Buitelaar JK, Beckmann CF. ICA-AROMA: A robust ICA-based strategy for removing motion artifacts from fMRI data. *NeuroImage*. 2015;112:267-277. doi:10.1016/j.neuroimage.2015.02.064
16. Salimi-Khorshidi G, Douaud G, Beckmann CF, Glasser MF, Griffanti L, Smith SM. Automatic denoising of functional MRI data: Combining independent component analysis and hierarchical fusion of classifiers. *NeuroImage*. 2014;90:449-468. doi:10.1016/j.neuroimage.2013.11.046
17. Satterthwaite TD, Wolf DH, Loughead J, et al. Impact of in-scanner head motion on multiple measures of functional connectivity: Relevance for studies of neurodevelopment in youth. *NeuroImage*. 2012;60(1):623-632. doi:10.1016/j.neuroimage.2011.12.063
18. Siegel JS, Mitra A, Laumann TO, et al. Data Quality Influences Observed Links Between Functional Connectivity and Behavior. *Cereb Cortex*. 2017;27(9):4492-4502. doi:10.1093/cercor/bhw253
19. Van Dijk KRA, Sabuncu MR, Buckner RL. The influence of head motion on intrinsic functional connectivity MRI. *NeuroImage*. 2012;59(1):431-438. doi:10.1016/j.neuroimage.2011.07.044
20. Yan CG, Cheung B, Kelly C, et al. A comprehensive assessment of regional variation in the impact of

head micromovements on functional connectomics. *NeuroImage*. 2013;76:183-201. doi:10.1016/j.neuroimage.2013.03.004
21. Casey BJ, Cannonier T, Conley MI, et al. The Adolescent Brain Cognitive Development (ABCD) study: Imaging acquisition across 21 sites. *Dev Cogn Neurosci*. 2018;32:43-54. doi:10.1016/j.dcn.2018.03.001
22. Greene DJ, Koller JM, Hampton JM, et al. Behavioral interventions for reducing head motion during MRI scans in children. *NeuroImage*. 2018;171:234-245. doi:10.1016/j.neuroimage.2018.01.023
23. Pagliaccio D, Luby J, Gaffrey M, et al. Anomalous functional brain activation following negative mood induction in children with pre-school onset major depression. *Dev Cogn Neurosci*. 2012;2(2):256-267. doi:10.1016/j.dcn.2011.11.008
24. Vanderwal T, Kelly C, Eilbott J, Mayes LC, Castellanos FX. Inscapes : A movie paradigm to improve compliance in functional magnetic resonance imaging. *NeuroImage*. 2015;122:222-232. doi:10.1016/j.neuroimage.2015.07.069
25. Fair DA, Miranda-Dominguez O, Snyder AZ, et al. Correction of respiratory artifacts in MRI head motion estimates. *NeuroImage*. 2020;208:116400. doi:10.1016/j.neuroimage.2019.116400
26. Friston KJ, Williams S, Howard R, Frackowiak RSJ, Turner R. Movement-Related effects in fMRI time-series: Movement Artifacts in fMRI. *Magn Reson Med*. 1996;35(3):346-355. doi:10.1002/mrm.1910350312
27. Power JD, Mitra A, Laumann TO, Snyder AZ, Schlaggar BL, Petersen SE. Methods to detect, characterize, and remove motion artifact in resting state fMRI. *NeuroImage*. 2014;84:320-341. doi:10.1016/j.neuroimage.2013.08.048
28. Power JD, Plitt M, Gotts SJ, et al. Ridding fMRI data of motion-related influences: Removal of signals with distinct spatial and physical bases in multiecho data. *Proc Natl Acad Sci*. 2018;115(9). doi:10.1073/pnas.1720985115
29. Satterthwaite TD, Elliott MA, Gerraty RT, et al. An improved framework for confound regression and filtering for control of motion artifact in the preprocessing of resting-state functional connectivity data. *NeuroImage*. 2013;64:240-256. doi:10.1016/j.neuroimage.2012.08.052
30. Tyszka JM, Kennedy DP, Paul LK, Adolphs R. Largely Typical Patterns of Resting-State Functional Connectivity in High-Functioning Adults with Autism. *Cereb Cortex*. 2014;24(7):1894-1905. doi:10.1093/cercor/bht040
31. Nebel MB, Lidstone DE, Wang L, Benkeser D, Mostofsky SH, Risk BB. Accounting for motion in resting-state fMRI: What part of the spectrum are we characterizing in autism spectrum disorder? *NeuroImage*. 2022;257:119296. doi:10.1016/j.neuroimage.2022.119296
32. Murphy K, Fox MD. Towards a consensus regarding global signal regression for resting state functional connectivity MRI. *NeuroImage*. 2017;154:169-173. doi:10.1016/j.neuroimage.2016.11.052
33. Gratton C, Dworetsky A, Coalson RS, et al. Removal of high frequency contamination from motion estimates in single-band fMRI saves data without biasing functional connectivity. *NeuroImage*. 2020;217:116866. doi:10.1016/j.neuroimage.2020.116866
34. Havsteen I, Ohlhues A, Madsen KH, Nybing JD, Christensen H, Christensen A. Are Movement Artifacts in Magnetic Resonance Imaging a Real Problem?—A Narrative Review. *Front Neurol*. 2017;8:232. doi:10.3389/fneur.2017.00232
35. Griffanti L, Douaud G, Bijsterbosch J, et al. Hand classification of fMRI ICA noise components. *NeuroImage*. 2017;154:188-205. doi:10.1016/j.neuroimage.2016.12.036
36. Alfaro-Almagro F, Jenkinson M, Bangerter NK, et al. Image processing and Quality Control for the first 10,000 brain imaging datasets from UK Biobank. *NeuroImage*. 2018;166:400-424. doi:10.1016/j.neuroimage.2017.10.034
37. Feczko E, Conan G, Marek S, et al. *Adolescent Brain Cognitive Development (ABCD) Community MRI Collection and Utilities*. Neuroscience; 2021. doi:10.1101/2021.07.09.451638
38. Glasser MF, Sotiropoulos SN, Wilson JA, et al. The minimal preprocessing pipelines for the Human Connectome Project. *NeuroImage*. 2013;80:105-124. doi:10.1016/j.neuroimage.2013.04.127
39. Hagler DJ, Hatton SeanN, Cornejo MD, et al. Image processing and analysis methods for the Adolescent Brain Cognitive Development Study. *NeuroImage*. 2019;202:116091. doi:10.1016/j.neuroimage.2019.116091
40. Power JD, Schlaggar BL, Petersen SE. Recent progress and outstanding issues in motion correction in

resting state fMRI. *NeuroImage*. 2015;105:536-551. doi:10.1016/j.neuroimage.2014.10.044
41. Phạm DĐ, McDonald DJ, Ding L, Nebel MB, Mejia AF. Less is more: balancing noise reduction and data retention in fMRI with data-driven scrubbing. *NeuroImage*. 2023;270:119972. doi:10.1016/j.neuroimage.2023.119972
42. Winkler AM, Webster MA, Brooks JC, Tracey I, Smith SM, Nichols TE. Non-parametric combination and related permutation tests for neuroimaging: NPC and Related Permutation Tests for Neuroimaging. *Hum Brain Mapp*. 2016;37(4):1486-1511. doi:10.1002/hbm.23115
43. Noble S, Mejia AF, Zalesky A, Scheinost D. Improving power in functional magnetic resonance imaging by moving beyond cluster-level inference. *Proc Natl Acad Sci*. 2022;119(32):e2203020119. doi:10.1073/pnas.2203020119
44. Jernigan TL, Brown SA. Introduction. *Dev Cogn Neurosci*. 2018;32:1-3. doi:10.1016/j.dcn.2018.02.002
45. Volkow ND, Koob GF, Croyle RT, et al. The conception of the ABCD study: From substance use to a broad NIH collaboration. *Dev Cogn Neurosci*. 2018;32:4-7. doi:10.1016/j.dcn.2017.10.002
46. Barch DM, Albaugh MD, Avenevoli S, et al. Demographic, physical and mental health assessments in the adolescent brain and cognitive development study: Rationale and description. *Dev Cogn Neurosci*. 2018;32:55-66. doi:10.1016/j.dcn.2017.10.010
47. Luciana M, Bjork JM, Nagel BJ, et al. Adolescent neurocognitive development and impacts of substance use: Overview of the adolescent brain cognitive development (ABCD) baseline neurocognition battery. *Dev Cogn Neurosci*. 2018;32:67-79. doi:10.1016/j.dcn.2018.02.006
48. Marek S, Tervo-Clemmens B, Calabro FJ, et al. Reproducible brain-wide association studies require thousands of individuals. *Nature*. 2022;603(7902):654-660. doi:10.1038/s41586-022-04492-9
49. Elam JS, Glasser MF, Harms MP, et al. The Human Connectome Project: A retrospective. *NeuroImage*. 2021;244:118543. doi:10.1016/j.neuroimage.2021.118543
50. Van Essen DC, Smith SM, Barch DM, Behrens TEJ, Yacoub E, Ugurbil K. The WU-Minn Human Connectome Project: An overview. *NeuroImage*. 2013;80:62-79. doi:10.1016/j.neuroimage.2013.05.041
51. Wechsler D, Pearson Education I, Psychological Corporation. *WISC-V: Wechsler Intelligence Scale for Children*. NCS Pearson, Inc. : PsychCorp; 2014.
52. Grummer-Strawn LM, Reinold C, Krebs NF, Centers for Disease Control and Prevention (CDC). Use of World Health Organization and CDC growth charts for children aged 0-59 months in the United States. *MMWR Recomm Rep Morb Mortal Wkly Rep Recomm Rep*. 2010;59(RR-9):1-15.
53. Laumann TO, Gordon EM, Adeyemo B, et al. Functional System and Areal Organization of a Highly Sampled Individual Human Brain. *Neuron*. 2015;87(3):657-670. doi:10.1016/j.neuron.2015.06.037
54. Stouffer SA, Suchman EA, Devinney LC, Star SA, Williams Jr. RM. *The American Soldier: Adjustment during Army Life. (Studies in Social Psychology in World War II), Vol. 1*. Princeton Univ. Press; 1949:xii, 599.
55. van Zwet WR, Oosterhoff J. On the Combination of Independent Test Statistics. *Ann Math Stat*. 1967;38(3):659-680.
56. Abreu R, Leal A, Figueiredo P. Identification of epileptic brain states by dynamic functional connectivity analysis of simultaneous EEG-fMRI: a dictionary learning approach. *Sci Rep*. 2019;9(1):638. doi:10.1038/s41598-018-36976-y
57. Couvy-Duchesne B, Blokland GAM, Hickie IB, et al. Heritability of head motion during resting state functional MRI in 462 healthy twins. *NeuroImage*. 2014;102:424-434. doi:10.1016/j.neuroimage.2014.08.010
58. Engelhardt LE, Roe MA, Juranek J, et al. Children's head motion during fMRI tasks is heritable and stable over time. *Dev Cogn Neurosci*. 2017;25:58-68. doi:10.1016/j.dcn.2017.01.011
59. Hodgson K, Poldrack RA, Curran JE, et al. Shared Genetic Factors Influence Head Motion During MRI and Body Mass Index. *Cereb Cortex*. Published online October 15, 2016:cercor;bhw321v1. doi:10.1093/cercor/bhw321
60. Zeng LL, Wang D, Fox MD, et al. Neurobiological basis of head motion in brain imaging. *Proc Natl Acad Sci*. 2014;111(16):6058-6062. doi:10.1073/pnas.1317424111
61. Jernigan T, Jernigan T. Adolescent Brain Cognitive Development Study (ABCD) - Annual Release 2.0. Published online 2019. doi:10.15154/1503209
62. Birn RM, Molloy EK, Patriat R, et al. The effect of scan length on the reliability of resting-state fMRI connectivity estimates. *NeuroImage*. 2013;83:550-558. doi:10.1016/j.neuroimage.2013.05.099
63. Jenkinson M, Beckmann CF, Behrens TEJ, Woolrich MW, Smith SM. FSL. *NeuroImage*.

2012;62(2):782-790. doi:10.1016/j.neuroimage.2011.09.015
64. Fischl B. Automatically Parcellating the Human Cerebral Cortex. *Cereb Cortex*. 2004;14(1):11-22. doi:10.1093/cercor/bhg087
65. Gordon EM, Laumann TO, Adeyemo B, Huckins JF, Kelley WM, Petersen SE. Generation and Evaluation of a Cortical Area Parcellation from Resting-State Correlations. *Cereb Cortex*. 2016;26(1):288-303. doi:10.1093/cercor/bhu239
66. Seitzman BA, Gratton C, Marek S, et al. A set of functionally-defined brain regions with improved representation of the subcortex and cerebellum. *NeuroImage*. 2020;206:116290. doi:10.1016/j.neuroimage.2019.116290
67. Raut RV, Mitra A, Snyder AZ, Raichle ME. On time delay estimation and sampling error in resting-state fMRI. *NeuroImage*. 2019;194:211-227. doi:10.1016/j.neuroimage.2019.03.020
68. Gratton C, Laumann TO, Nielsen AN, et al. Functional Brain Networks Are Dominated by Stable Group and Individual Factors, Not Cognitive or Daily Variation. *Neuron*. 2018;98(2):439-452.e5. doi:10.1016/j.neuron.2018.03.035
69. Guan S, Jiang R, Bian H, et al. The Profiles of Non-stationarity and Non-linearity in the Time Series of Resting-State Brain Networks. *Front Neurosci*. 2020;14:493. doi:10.3389/fnins.2020.00493
70. Politis DN, Romano JP. The Stationary Bootstrap. *J Am Stat Assoc*. 1994;89(428):1303-1313. doi:10.1080/01621459.1994.10476870
71. Ellis CT, Baldassano C, Schapiro AC, Cai MB, Cohen JD. Facilitating open-science with realistic fMRI simulation: validation and application. *PeerJ*. 2020;8:e8564. doi:10.7717/peerj.8564
72. Smyser CD, Snyder AZ, Neil JJ. Functional connectivity MRI in infants: Exploration of the functional organization of the developing brain. *NeuroImage*. 2011;56(3):1437-1452. doi:10.1016/j.neuroimage.2011.02.073
73. Afyouni S, Nichols TE. Insight and inference for DVARS. *NeuroImage*. 2018;172:291-312. doi:10.1016/j.neuroimage.2017.12.098
74. Smith SM, Beckmann CF, Andersson J, et al. Resting-state fMRI in the Human Connectome Project. *NeuroImage*. 2013;80:144-168. doi:10.1016/j.neuroimage.2013.05.039
