# Supplementary material for: Motion impact score for detecting spurious brain-behavior associations
Source: Nat Commun. 2025 Sep 29;16:8614. doi: 10.1038/s41467-025-63661-2 (PMC12479937; doi:10.1038/s41467-025-63661-2)
Supplement: Supplementary file 2 — Description of Additional Supplementary Files [file 41467_2025_63661_MOESM2_ESM.pdf]

Description of Additional Supplementary Files

Supplementary Data 1: Relationship between selected variables in the ABCD study with head motion (framewise displacement, FD) averaged over each participant's resting state fMRI scans. The SHAMAN (omnibus) p-values and motion scores (omnibus Stouffer's Z-scores) are also given for overestimation scores, underestimation scores, and impact from both/either type of score. Results in this table reflect data after motion-reduction with ABCD-BIDS and without additional motion censoring.

Supplementary Data 2: Significance (p-values) of motion impact score for selected variables in the ABCD study after motion-reduction with ABCD-BIDS and with (varying from left to right) no motion censoring, censoring at framewise displacement (FD) < 0.3 mm, FD < 0.2, and FD < 0.1. Omnibus p-values are given for overestimation scores, underestimation scores, and impact from both/either type of score. The omnibus p-values account for multiple comparisons across edges, but they are not corrected for the multiple comparisons across the 45 different traits shown.

Supplementary Data 3: Motion impact scores (omnibus Stouffer's Z) for selected variables in the ABCD study after motion-reduction with ABCD-BIDS and with (varying from left to right) no motion censoring, censoring at framewise displacement (FD) < 0.3 mm, FD < 0.2, and FD < 0.1. Higher scores correspond to a greater impact of motion. Motion overestimation scores, underestimation scores, and overall impact (both/either over/underestimation) are shown separately.

Supplementary Data 4: Sampling bias at different levels of motion censoring. The percentage difference in sample mean (compared to no censoring) is shown for selected variables in ABCD after censoring at a framewise displacement (FD) of 0.3, 0.2, or < 0.1 mm. Two variables (gender and number of MRI runs completed) are biased by more than 1% at FD < 0.2 mm, and 11 variables are biased by > 1% at FD < 0.1 mm.

Supplementary Data 5: Comparison between using all fMRI frames and clamping to 600 frames per participant. The 600 frames were chosen randomly without replacement. There

was no motion censoring. Motion impact scores that were significant ($p < 0.05$) using all frames but became not-significant ($p > 0.05$) with clamping to 600 frames are highlighted in red. Motion impact scores that were significant only with clamping to 600 frames are highlighted in blue.

Supplementary Data 6: Relationship between selected variables in the HCP study with head motion (framewise displacement, FD) averaged over each participant's resting state fMRI scans. The SHAMAN (omnibus) p-values and motion scores (omnibus Stouffer's Z-scores) are also given for overestimation scores, underestimation scores, and impact from both/either type of score. Results in this table reflect data after motion-reduction and without additional motion censoring.

Supplementary Data 7: Significance (p-values) of motion impact score for selected variables in the HCP study after motion-reduction and with (varying from left to right) no motion censoring, censoring at framewise displacement (FD) < 0.3 mm, FD < 0.2, and FD < 0.1. Omnibus p-values are given for overestimation scores, underestimation scores, and impact from both/either type of score. The omnibus p-values account for multiple comparisons across edges, but they are not corrected for the multiple comparisons across the 76 different traits shown.

Supplementary Data 8: Motion impact scores (omnibus Stouffer's Z) for selected variables in the HCP study after motion-reduction and with (varying from left to right) no motion censoring, censoring at framewise displacement (FD) < 0.3 mm, FD < 0.2, and FD < 0.1. Higher scores correspond to a greater impact of motion. Motion overestimation scores, underestimation scores, and overall impact (both/either over/underestimation) are shown separately.

Supplementary Data 9: Relationship between selected variables in the ABCD study with head motion (DVARS) averaged over each participant's resting state fMRI scans. The SHAMAN (omnibus) p-values and motion scores (omnibus Stouffer's Z-scores) are also given for overestimation scores, underestimation scores, and impact from both/either type of

score. Results in this table reflect data after motion-reduction with ABCD-BIDS and without additional motion censoring.

Supplementary Data 10: Significance (p-values) of motion impact score for selected variables in the ABCD study after motion-reduction with ABCD-BIDS and with (varying from left to right) no motion censoring, censoring at DVARS < 200, and censoring at a DVARS p-value > 0.05. Omnibus p-values are given for overestimation scores, underestimation scores, and impact from both/either type of score. The omnibus p-values account for multiple comparisons across edges, but they are not corrected for the multiple comparisons across the 45 different traits shown.

Supplementary Data 11: Motion impact scores (omnibus Stouffer's Z) for selected variables in the ABCD study after motion-reduction with ABCD-BIDS and with (varying from left to right) no motion censoring, censoring at DVARS < 200, and censoring at a DVARS p-value > 0.05. Higher scores correspond to a greater impact of motion. Motion overestimation scores, underestimation scores, and overall impact (both/either over/underestimation) are shown separately.

Supplementary Data 12: Relationship between selected variables in the HCP study with head motion (DVARS) averaged over each participant's resting state fMRI scans. The SHAMAN (omnibus) p-values and motion scores (omnibus Stouffer's Z-scores) are also given for overestimation scores, underestimation scores, and impact from both/either type of score. Results in this table reflect data after motion-reduction and without additional motion censoring.

Supplementary Data 13: Significance (p-values) of motion impact score for selected variables in the HCP study after motion-reduction and with (varying from left to right) no motion censoring, censoring at DVARS < 200, and censoring at a DVARS p-value > 0.05. Omnibus p-values are given for overestimation scores, underestimation scores, and impact from both/either type of score. The omnibus p-values account for multiple comparisons across edges, but they are not corrected for the multiple comparisons across the 76 different traits shown.

Supplementary Data 14: Motion impact scores (omnibus Stouffer's Z) for selected variables in the HCP study after motion-reduction and with (varying from left to right) no motion censoring, censoring at DVARS < 200, and censoring at a DVARS p-value > 0.05. Higher scores correspond to a greater impact of motion. Motion overestimation scores, underestimation scores, and overall impact (both/either over/underestimation) are shown separately.
