# Supplementary material for: Motion impact score for detecting spurious brain-behavior associations
Source: Nat Commun. 2025 Sep 29;16:8614. doi: 10.1038/s41467-025-63661-2 (PMC12479937; doi:10.1038/s41467-025-63661-2)
Supplement: Supplementary file 17 — Reporting Summary [file 41467_2025_63661_MOESM17_ESM.pdf]

nature portfolio

Corresponding author(s): Benjamin P Kay

Last updated by author(s): Jul 16, 2025

Reporting Summary

Nature Portfolio wishes to improve the reproducibility of the work that we publish. This form provides structure for consistency and transparency in reporting. For further information on Nature Portfolio policies, see our Editorial Policies and the Editorial Policy Checklist.

Statistics

For all statistical analyses, confirm that the following items are present in the figure legend, table legend, main text, or Methods section.

n/a	Confirmed	
☐	☒	The exact sample size (*n*) for each experimental group/condition, given as a discrete number and unit of measurement
☐	☒	A statement on whether measurements were taken from distinct samples or whether the same sample was measured repeatedly
☐	☒	The statistical test(s) used AND whether they are one- or two-sided<br>*Only common tests should be described solely by name; describe more complex techniques in the Methods section.*
☐	☒	A description of all covariates tested
☐	☒	A description of any assumptions or corrections, such as tests of normality and adjustment for multiple comparisons
☐	☒	A full description of the statistical parameters including central tendency (e.g. means) or other basic estimates (e.g. regression coefficient) AND variation (e.g. standard deviation) or associated estimates of uncertainty (e.g. confidence intervals)
☐	☒	For null hypothesis testing, the test statistic (e.g. *F*, *t*, *r*) with confidence intervals, effect sizes, degrees of freedom and *P* value noted<br>*Give P values as exact values whenever suitable.*
☐	☒	For Bayesian analysis, information on the choice of priors and Markov chain Monte Carlo settings
☐	☒	For hierarchical and complex designs, identification of the appropriate level for tests and full reporting of outcomes
☐	☒	Estimates of effect sizes (e.g. Cohen's *d*, Pearson's *r*), indicating how they were calculated

Our web collection on statistics for biologists contains articles on many of the points above.

Software and code

Policy information about availability of computer code

Data collection	ABCD data are from the ABCD 2.0 release and were processed using the ABCD-BIDS pipeline.
Data analysis	Analysis was performed using Matlab R2022a.

For manuscripts utilizing custom algorithms or software that are central to the research but not yet described in published literature, software must be made available to editors and reviewers. We strongly encourage code deposition in a community repository (e.g. GitHub). See the Nature Portfolio guidelines for submitting code & software for further information.

Data

Policy information about availability of data

All manuscripts must include a data availability statement. This statement should provide the following information, where applicable:

- Accession codes, unique identifiers, or web links for publicly available datasets
- A description of any restrictions on data availability
- For clinical datasets or third party data, please ensure that the statement adheres to our policy

The ABCD 2.0 release data are freely available from the NIMH Data Archive (NDA). Users must approve a data use agreement prior to downloading the data.

Research involving human participants, their data, or biological material

Policy information about studies with human participants or human data. See also policy information about sex, gender (identity/presentation), and sexual orientation and race, ethnicity and racism.

Reporting on sex and gender	We report on the impact of motion on the trait-FC relationship of biological sex assigned at birth (parent report).
Reporting on race, ethnicity, or other socially relevant groupings	The ABCD data are from a national consortium of 21 sites. Participant selection was intented to produce an ethnically and racially diverse sample. The analyses in this manuscript do not explicitly investigate the effects of race or ethnicity.
Population characteristics	Please refer to the population characteristics of the ABCD Study.
Recruitment	Please refer to the description of the ABCD Study.
Ethics oversight	The ABDC Study obtained centralized IRB approval from the University of California, San Diego. Each of the 21 study sites also obtained local IRB approval.

Note that full information on the approval of the study protocol must also be provided in the manuscript.

Field-specific reporting

Please select the one below that is the best fit for your research. If you are not sure, read the appropriate sections before making your selection.

☒ Life sciences ☐ Behavioural & social sciences ☐ Ecological, evolutionary & environmental sciences

For a reference copy of the document with all sections, see nature.com/documents/nr-reporting-summary-flat.pdf

Life sciences study design

All studies must disclose on these points even when the disclosure is negative.

Sample size	The sample size was n = 7,270 participants. The sample size was determined from the number of the original 11,572 participants in the ABCD Study with complete data.
Data exclusions	Participants with missing data (e.g. declined to provide demographic information), missing/poor quality anatomical MRI scans, or less than 8 minutes of resting-state fMRI data were excluded.
Replication	A replication of the SHAMAN method and key findings using data from the Human Connectome Project is described in the Supplementary Material.
Randomization	N/A
Blinding	N/A

Reporting for specific materials, systems and methods

We require information from authors about some types of materials, experimental systems and methods used in many studies. Here, indicate whether each material, system or method listed is relevant to your study. If you are not sure if a list item applies to your research, read the appropriate section before selecting a response.

Materials & experimental systems

n/a	Involved in the study	
☒	☐	Antibodies
☒	☐	Eukaryotic cell lines
☒	☐	Palaeontology and archaeology
☒	☐	Animals and other organisms
☒	☐	Clinical data
☒	☐	Dual use research of concern
☒	☐	Plants

Methods

n/a	Involved in the study	
☒	☐	ChIP-seq
☒	☐	Flow cytometry
☐	☒	MRI-based neuroimaging

Plants

Seed stocks	N/A
Novel plant genotypes	N/A
Authentication	N/A

Magnetic resonance imaging

Experimental design

Design type	Observational/Cross-sectional
Design specifications	Up to four 5-minute resting-state fMRI scans per participant.
Behavioral performance measures	See methods; these include in-scanner head motion and out-of-scanner CBCL and NIH Toolbox performance.

Acquisition

Imaging type(s)	T1, T2, and fMRI/BOLD.
Field strength	3 Tesla
Sequence & imaging parameters	TR = 800 ms, specific parameters vary by scanner manufacturer, see published methods for ABCD Study.
Area of acquisition	Whole brain

Diffusion MRI ☐ Used ☒ Not used

Preprocessing

Preprocessing software	Containerized ABCD-BIDS pipeline including components from AFNI, FSL, and ANTS.
Normalization	Volumetric and surface based registration
Normalization template	MNI and FSL
Noise and artifact removal	ABCD-BIDS pipeline including motion de-spiking and global signal regression
Volume censoring	Based on framewise displacement, varying from no censoring to 0.3, 0.2, or 0.1 mm cutoffs. DVARS in Supplementary Material.

Statistical modeling & inference

Model type and settings	Mass-univariate model with non-parametric permutation testing and omnibus combining (Stouffer's Z statistic) to account for multiple comparisons.
Effect(s) tested	Relationship between traits (e.g. NIH toolbox performance) and resting-state fMRI connectivity (atanh/Fisher-Z transformed correlation).

Specify type of analysis: ☐ Whole brain ☐ ROI-based ☒ Both

Anatomical location(s): Whole-brain analysis using Gordon-Laumann-Sietzmann 394 parcels.

Statistic type for inference (See Eklund et al. 2016)	Edge-wise
Correction	Non-parametric combining to control family wise error rate (multiple comparisons).

Models & analysis

n/a	Involved in the study
☐	☒ Functional and/or effective connectivity
☒	☐ Graph analysis
☒	☐ Multivariate modeling or predictive analysis

Functional and/or effective connectivity: Fisher-Z (atanh) transformed correlation
